# Integrative analysis of epilepsy-associated genes reveals expression-phenotype correlations

**DOI:** 10.1038/s41598-024-53494-2

**Published:** 2024-02-13

**Authors:** Wanhao Chi, Evangelos Kiskinis

**Affiliations:** 1 The Ken & Ruth Davee Department of Neurology, Feinberg School of Medicine, Northwestern University, Chicago, IL 60611 USA; 2 Simpson Querrey Institute, Northwestern University, Chicago, IL 60611 USA; 3 Department of Neuroscience, Feinberg School of Medicine, Northwestern University, Chicago, IL 60611 USA

**Keywords:** Engineering, Civil engineering

## Abstract

Epilepsy is a highly prevalent neurological disorder characterized by recurrent seizures. Patients exhibit broad genetic, molecular, and clinical diversity involving mild to severe comorbidities. The factors that contribute to this phenotypic diversity remain unclear. Here we used publicly available datasets to systematically interrogate the expression pattern of 230 epilepsy-associated genes across human tissues, developmental stages, and central nervous system (CNS) cellular subtypes. We grouped genes based on their curated phenotypes into 3 broad classes: core epilepsy genes (CEG), where seizures are the dominant phenotype, developmental and epileptic encephalopathy genes (DEEG) that are associated with developmental and epileptic encephalopathy, and seizure-related genes (SRG), which are characterized by the presence of seizures and gross brain malformations. We find that compared to the other two groups of genes, DEEGs are highly expressed within the adult CNS, exhibit the highest and most dynamic expression in various brain regions across development, and are significantly enriched in GABAergic neurons. Our analysis provides an overview of the expression pattern of epilepsy-associated genes with spatiotemporal resolution and establishes a broad expression-phenotype correlation in epilepsy.

## Introduction

Epilepsy constitutes one of the most common neurological disorders estimated to affect 70 million people worldwide^1^. It is primarily characterized by recurrent and unprovoked seizures in the brain^2^. People with epilepsy often exhibit developmental impairments and other comorbidities^3^. The pathophysiological mechanisms underlying the phenotypic diversity associated with epilepsy remain unclear.

Genetic predisposition plays an important role in epilepsy^4^, and several hundred epilepsy-associated genes have been identified^5,6^. These genes have diverse molecular functions and are implicated in different disease subtypes. One intriguing question is whether the diverse clinical manifestation seen in individuals with epilepsy correlates with the spatiotemporal expression of associated genes. This question has not been systematically addressed, partially due to the lack of extensive human gene expression datasets. In recent years, several large and publicly-accessible gene expression data have been assembled, including complete human tissue expression data from the Genotype-Tissue Expression (GTEx) project^7^ and brain developmental and cell type-specific expression data from the Allen Brain Institute^8,9^. The availability of these extensive and high-quality datasets makes it possible to investigate gene expression-phenotype correlations.

Here we examined the expression pattern of epilepsy-associated genes at the tissue, developmental stage, and single-cell levels. We generated a list of 247 epilepsy-associated genes from the Online Mendelian Inheritance in Man (OMIM)^10^ catalog that records gene-disease relationships, and categorized these genes into three broad groups based on their associated phenotypes: (a) core epilepsy genes (CEG), which are associated with various subtypes of seizures as the core phenotype; (b) developmental and epileptic encephalopathy genes (DEEG), which are characterized by early onset developmental and epileptic encephalopathy as the key phenotype; (c) seizure-related genes (SRG), which are associated with seizures and gross brain malformations. We further cross-referenced these groups of genes with two recent studies that have reviewed and curated genes associated with epilepsy^6,11^. To examine their expression, we obtained bulk RNA sequencing (RNA-seq) and single nucleus RNA-seq datasets from GTEx^7^ and the Allen Brain Atlas^8,9^ and performed hierarchical clustering analyses. We found that CEGs and DEEGs exhibit differential expression between central nervous system (CNS) and non-CNS tissues, that DEEGs are expressed 2-to-3-fold higher than CEGs or SRGs in various brain regions across development, and that expression of individual epilepsy-associated genes is largely comparable between cortical GABAergic and glutamatergic neurons. The high expression level of DEEGs during development may contribute to neurodevelopmental impairments associated with this class of genes. Taken together, our analysis provides an overview of the expression pattern of epilepsy-associated genes with spatiotemporal resolution and establishes a broad expression-phenotype correlation in epilepsy.

## Results

### Selection and categorization of epilepsy-associated genes into three distinct groups based on phenotypes curated in OMIM

**Figure 1:**
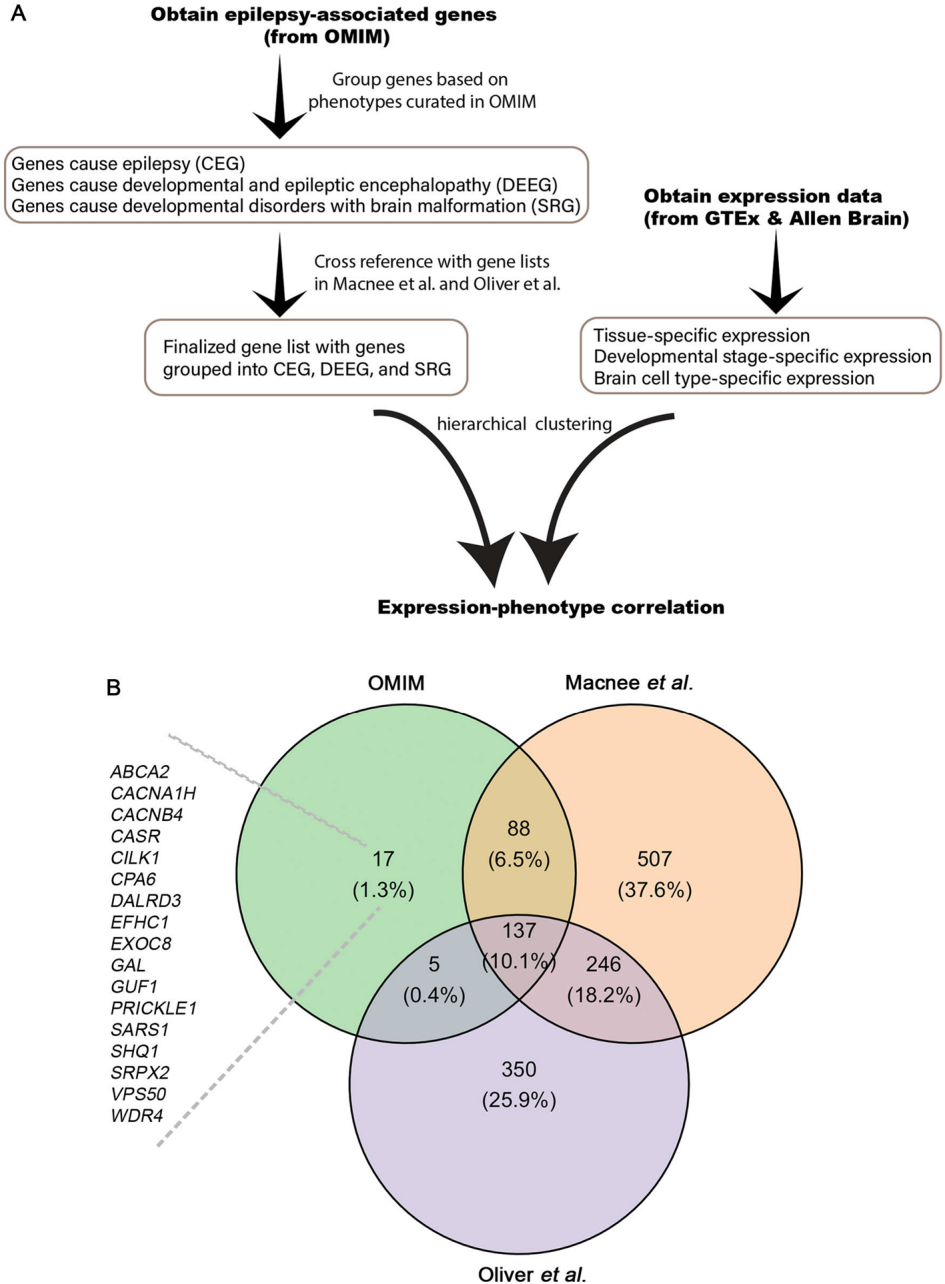
An overview of the datasets and analysis pipeline. (**A**) Flow of the study. (**B**) A Venn diagram of genes lists from OMIM, Macnee *et al.*^6^, and Oliver *et al.*^11^. Gene number and percentage in each category are shown.

We obtained 247 epilepsy-associated genes from OMIM^10^, an Online Catalog of Human Genes and Genetic Disorders that curates both genes and mutations with related clinical phenotypes in patients by querying the following terms: [E/e]pilepsy, [E/e]pilepsies, [E/e]pileptic, [S/s]eizure, and [S/s]eizures (Supplemental Table 1). We manually categorized these genes into three groups based on the curated phenotypes. Genes with ’*Developmental and epileptic encephalopathy*’ listed in the phenotype column were grouped into the category of developmental and epileptic encephalopathy gene (DEEG), genes with ’*Epilepsy*’ listed in the phenotype column were grouped into the category of core-epilepsy gene (CEG), and genes that didn’t list these phenotypes were grouped into the category of seizure-related gene (SRG). SRGs are typically associated with developmental defects and brain malformation, according to the phenotypes cureated in OMIM and ClinVar (Fig.  1A, Supplemental Table 1). To ensure the validity of our gene selection, we cross-referenced them with two recent studies by Macnee *et al.*^6^ and Oliver *et al.*^11^, in which a total of 738 and 978 genes were curated as epilepsy-associated genes, respectively. It is noteworthy that only 383 genes are shared between the two datasets, accounting for 28.7% of the total number (Fig.  1B). When we cross-referenced the OMIM gene list with these two datasets, we found that 17 genes in the OMIM gene list that were not included in either of these studies. We therefore excluded these 17 genes in our final analyses (Fig. 1B).

**Figure 2:**
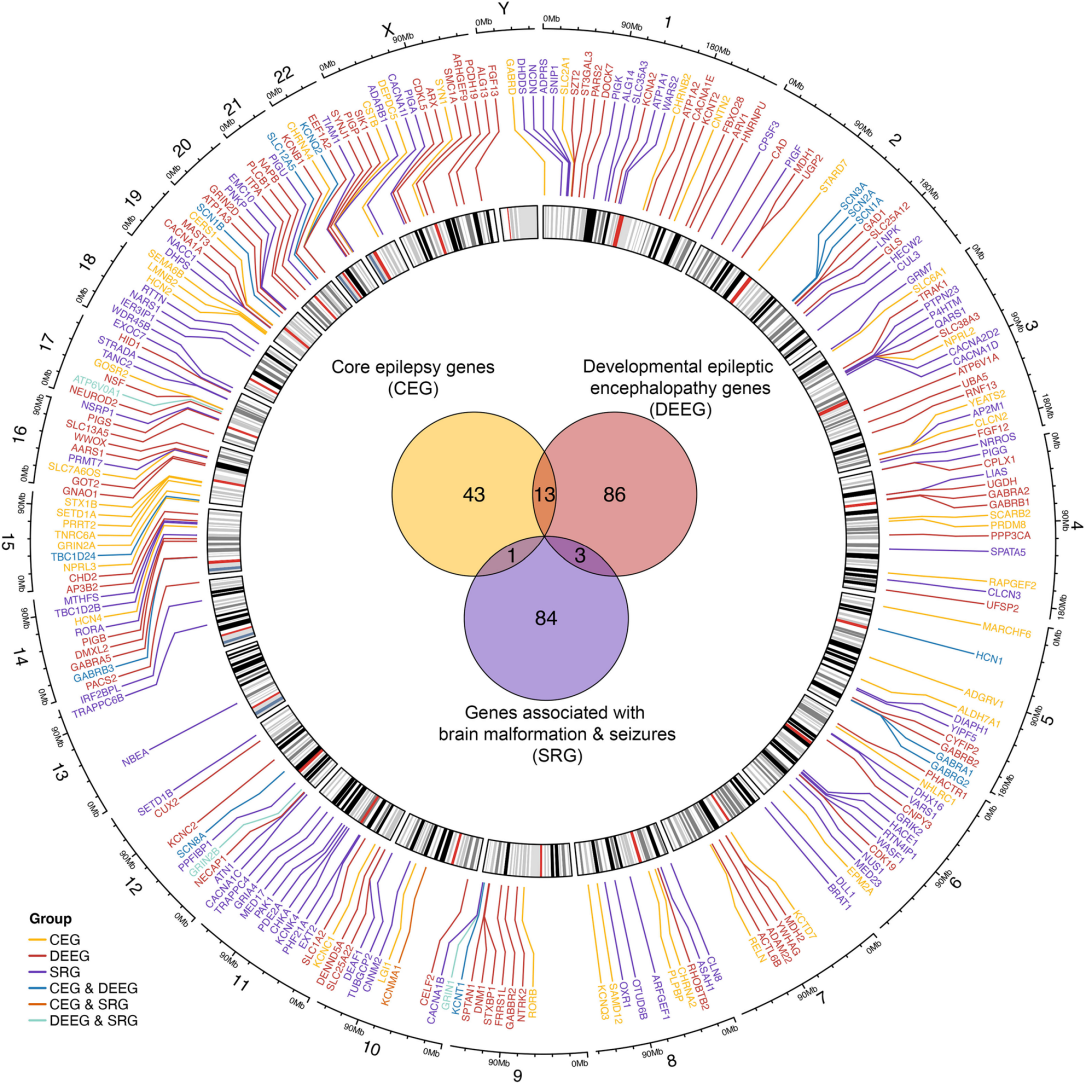
Genomic profile of epilepsy-associated genes. A Circos plot representing the genomic location of epilepsy-associated genes. Epilepsy-associated genes are color-coded based on phenotypes identified in patients carrying mutations in corresponding genes. Inside the Circos is a Venn diagram that shows the number of genes in each group.

**Figure 3:**
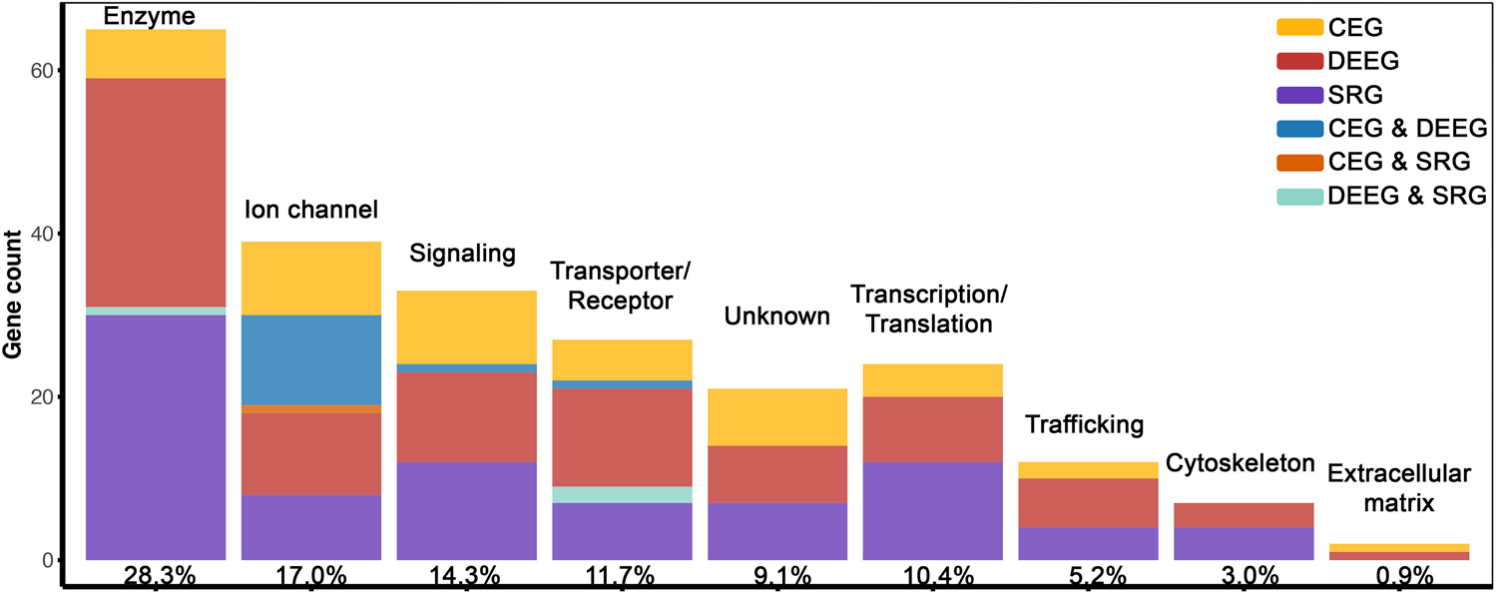
Functional classification of epilepsy-associated genes. Protein Class categories for 230 epilepsy-associated genes are based on PANTHER^53^ The percent of each category relative to all genes is shown on the X-axis.

The final gene list contains 230 genes (Supplemental Table 1). While most genes are unique within each group, we found thirteen genes shared by CEG and DEEG: *GABRA1*, *GABRB3*, *SCN3A*, *SLC12A5*, *TBC1D24*, *SCN1A*, *GABRG2*, *SCN1B*, *HCN1*, *SCN2A*, *SCN8A*, *KCNQ2*, and *KCNT1*. Additionally, *KCNMA1* is associated with both CEG and SRG, while *ATP6V0A1*, *GRIN1*, and *GRIN2B* are associated with both DEEG and SRG (Fig. 2). To minimize the ambiguity, these genes were excluded in the downstream group expression analyses. Instead, these were considered individually and their expression data is presented in Supplemental Figs. 1–3.

Epilepsy-associated genes are encoded relatively uniformly across the genome (Fig. 2). Each chromosome contains multiple genes except for chromosomes 13 and Y, which have one and none, respectively. The absence of epilepsy-associated genes on chromosome Y is likely attributed to the low number of total genes (200), with the average ratio of epilepsy genes relative to the total number of genes for all other chromosomes being 0.68% (Supplemental Table 1). Notably, epilepsy-associated genes are enriched for various functional categories, including “enzyme” (28.3%), “ion channel” (17.0%), “signaling” (14.3%), “transporter/receptor” (11.7%),“transcription/translation” (10.4%), “trafficking” (5.2%), “cytoskeleton” (3.0%), and “extracellular matrix” (0.9%), while several genes remain uncharacterized (9.1%) (Fig. 3). The diverse protein functions of epilepsy-associated genes suggest that neuronal activity in the brain is intricately modulated.

### CEGs and DEEGs exhibit differential expression between CNS and non-CNS tissues

**Figure 4:**
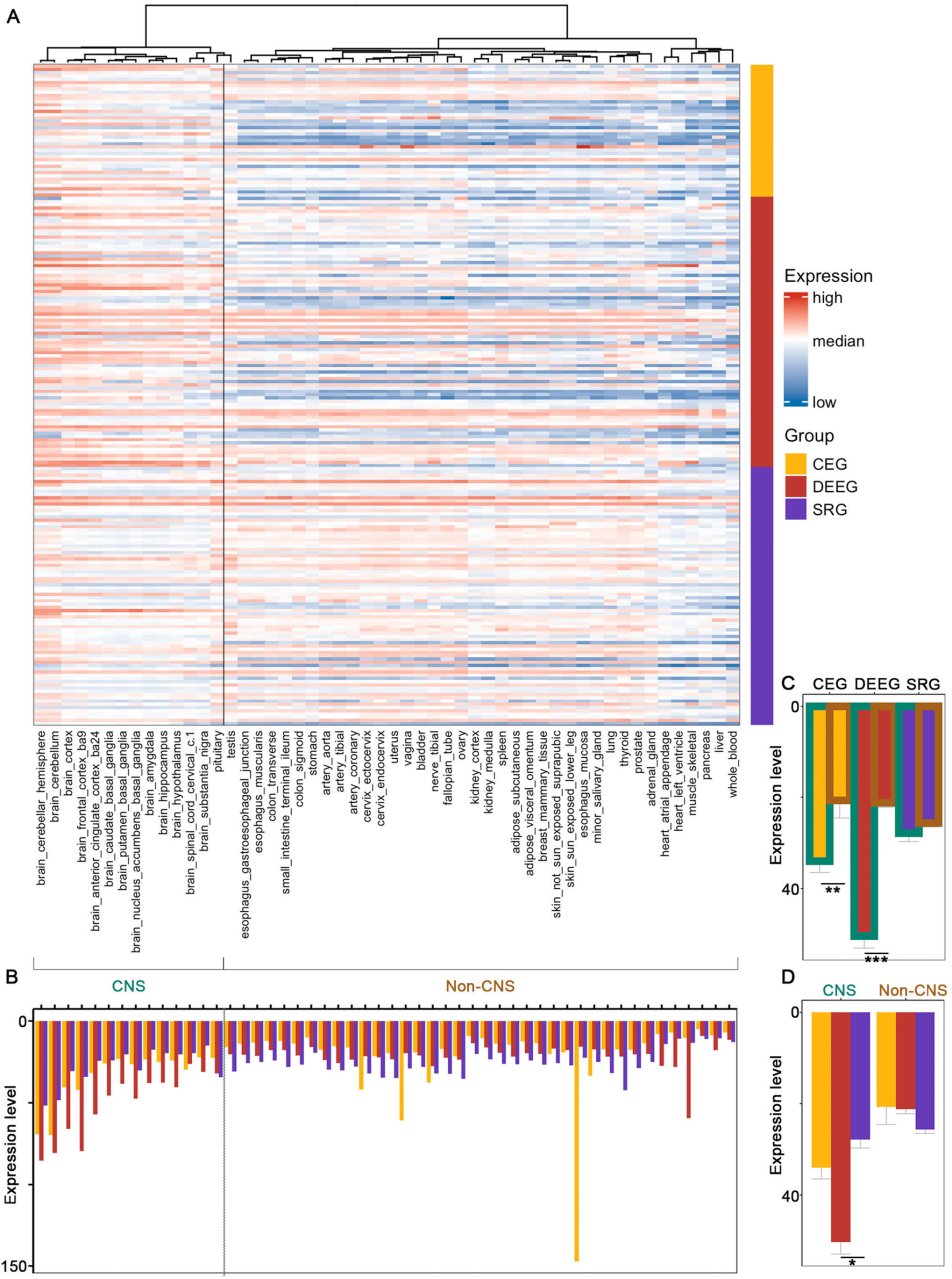
Expression of the three groups of epilepsy-associated genes in human tissues. (**A**) A heatmap for hierarchical clustering. (**B**) Averaged expression of all the genes in each group across different tissues. (**C**, **D**) Averaged expression of all the genes in each group in CNS versus non-CNS tissues. *$p<$ 0.05, **$p<$ 0.01, ***$p<$ 0.001, Wilcoxon signed rank sum test with Benjamini-Hochberg post hoc. Bulk RNA-seq expression data are from GTEx.

To assess the expression pattern for epilepsy-associated genes, we first obtained tissue-specific expression data from GTEx^7^, which includes 14 CNS tissues and 38 non-CNS regions. We performed hierarchical clustering analyses across all genes and samples and found a clear separation between CNS (and testis) and non-CNS tissues (Fig. 4A, Supplemental Fig. 4). In terms of epilepsy gene groups, CEGs and DEEGs showed significantly elevated averaged expression in CNS compared to non-CNS tissues (Fig. 4B-C). Within CNS tissues, DEEGs have the highest expression level, although the difference between DEEGs and CEGs is not statistically significant (Fig.  4D). It is noteworthy that the expression dataset used here is generated from adult tissues, and it is unknown whether similar expression patterns for these three groups of genes persist in fetal tissues. Lastly, individual genes identified as major contributors to epilepsy^6^, such as *SCN1A*, *SCN2A* and *KCNQ2* exhibited diverse expression within regions of the brain (Supplemental Fig. 1).

### DEEGs exhibit high expression across development

**Figure 5:**
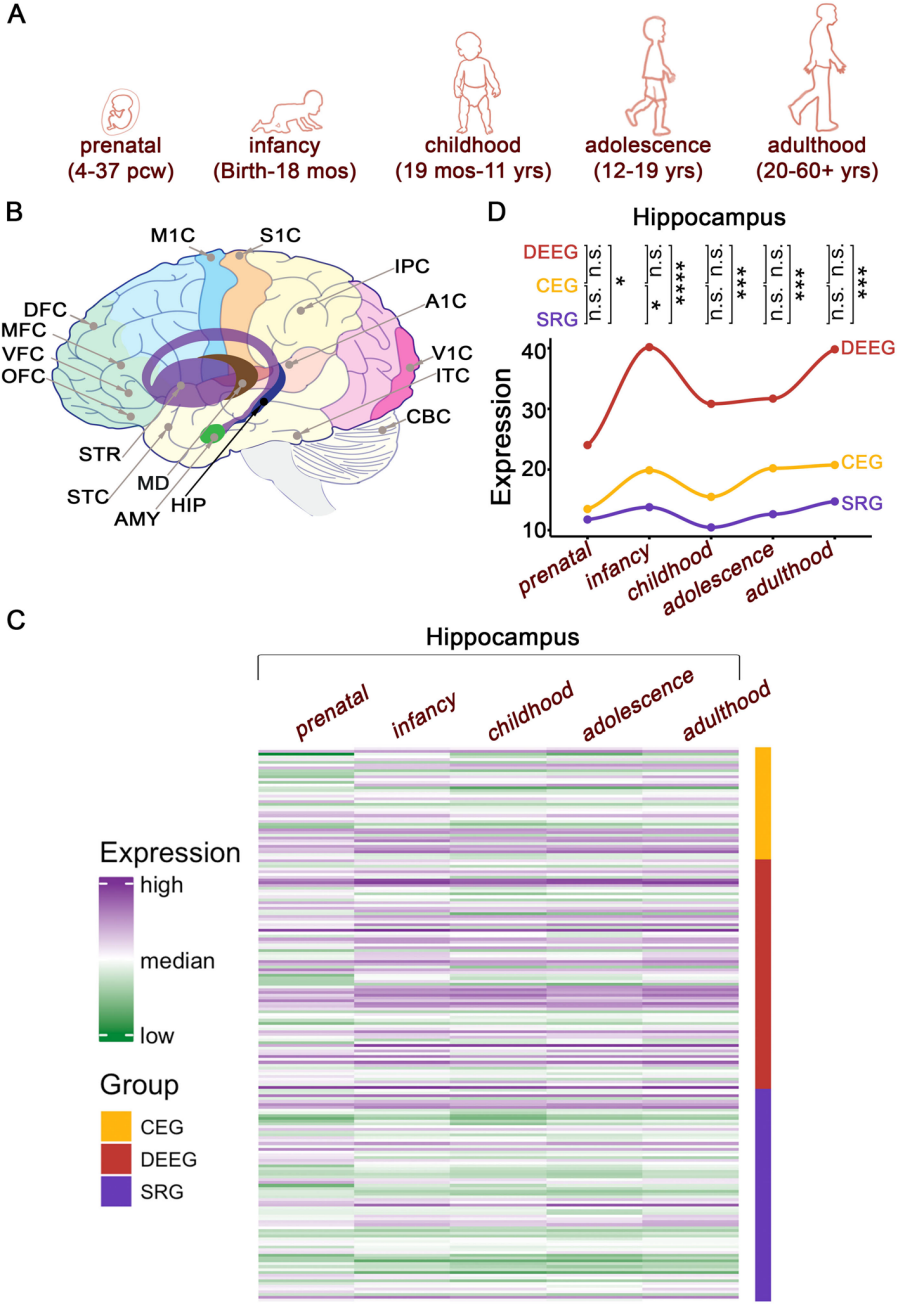
Expression of epilepsy-associated genes in the developing brain. (**A**) A cartoon presentation of human development with respective ages. (**B**) Brain sub-regions included in the analyses. A1C: primary auditory cortex; AMY: amygdala; CBC: cerebellar cortex; DFC: dorsolateral prefrontal cortex; HIP: hippocampus; IPC: posteroventral parietal cortex; ITC: inferolateral temporal cortex; M1C: primary motor cortex; MD: Mediodorsal nucleus of thalamus; MFC: anterior cingulate cortex; OFC: orbital frontal cortex; S1C: primary somatosensory cortex; STC: posterior superior temporal cortex; STR: striatum; V1C: primary visual cortex; VFC: ventrolateral prefrontal cortex. (**C**) Heatmap from hippocampus. (**D**) Averaged expression of all the genes within each group across development in the hippocampus. n.s. $p>$ 0.05, ***$p<$ 0.001, ****$p<$ 0.0001, Wilcoxon signed rank sum test with Benjamini-Hochberg post hoc. See Supplemental Figs. 6–7 for other regions. Bulk RNA-seq data are from Allen BrainSpan.

Given that DEE patients exhibit neurodevelopmental deficits, we next examined the developmental expression pattern of epilepsy-associated genes in the brain. We obtained RNA-seq data from different stages of human development, ranging from 4 post-conception weeks (PCW) to 60+ years, in 26 brain sub-regions generated by the Allen Brain Institute^9^ (Fig.  5A,B). For clarity, we grouped the datasets into one of five developmental stages: prenatal, infancy, childhood, adolescence, and adulthood (Fig.  5A). As a group, DEEGs exhibited significantly higher expression levels (two-three-fold) relative to SRGs within the hippocampus, as well as greater variation during development, rising from prenatal stages to infancy, normalizing during childhood, and then slowly increasing again in adolescence and adulthood (Fig.  5C,D; Supplemental Fig. 5). Close examination of individual gene expression across the five developmental periods within the hippocampus revealed that on average both DEEGs and CEGs transition into higher expression levels during the shift from prenatal to infancy, while SRGs were the least dynamic (Fig.  5D). These patterns were similarly observed across the other 15 brain regions we examined with minor variations (Supplemental Figs. 6–7).

### Expression of epilepsy-associated genes in neurons does not correlate with phenotypes

We further examined the expression of the three groups of epilepsy-associated genes in brain-specific cell types. We utilized an extensive set of single-nucleus human RNA-seq data from the Allen Brain Institute^8^, which contains sets from GABAergic and glutamatergic neurons and 4 types of non-neuronal cells: astrocytes, oligodendrocyte progenitor cells (OPCs), oligodendrocytes, and microglia. Within each neuronal cell type, individual cells are further classified into 26 subtypes of glutamatergic neurons and 19 subtypes of GABAergic interneurons based on the expression of specific molecular markers.

**Figure 6:**
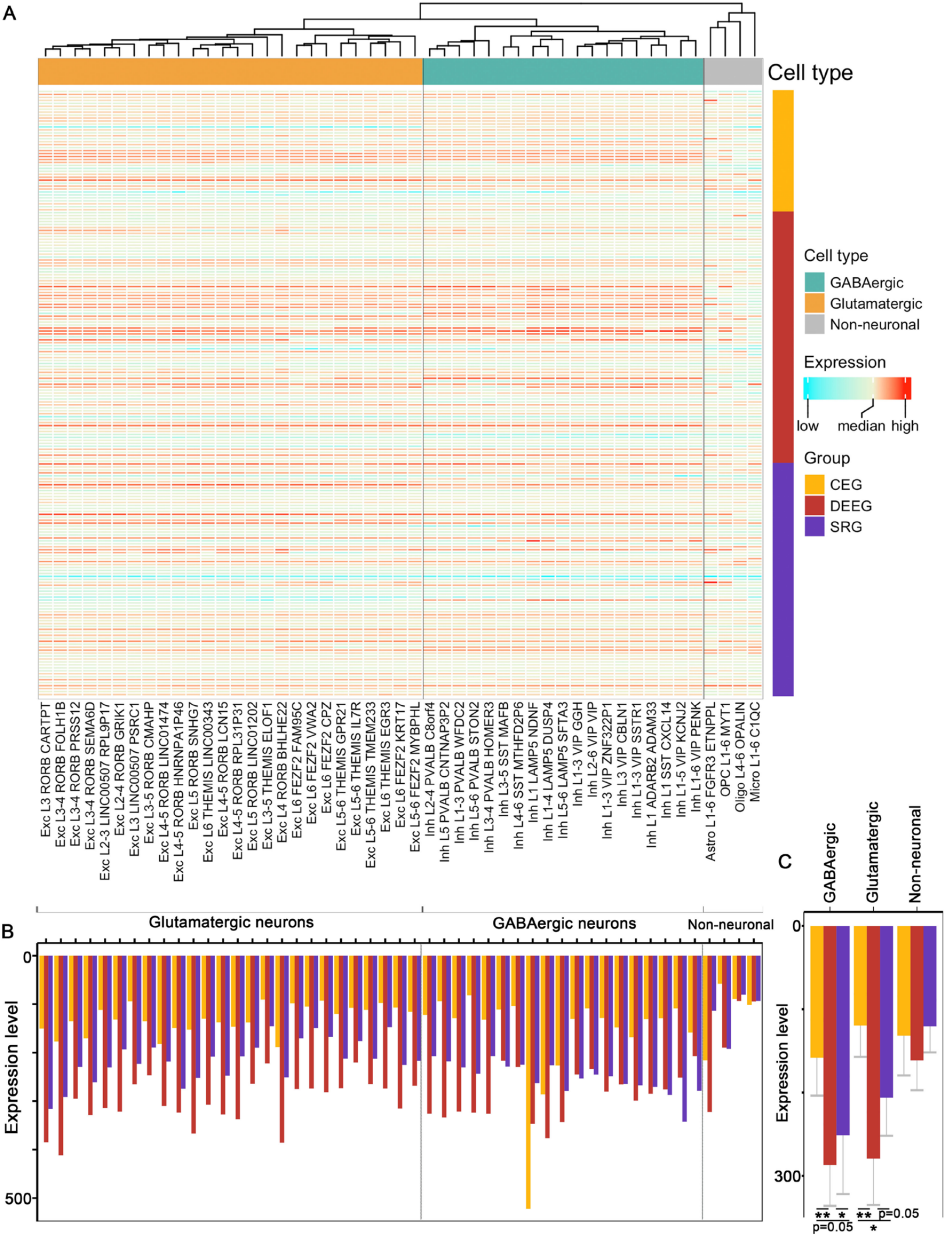
Expression of epilepsy-associated genes in different brain cell types. (**A**) A heatmap for hierarchical clustering across cell types. (**B**) Averaged expression of all the genes within each group across different cell type clusters. (**C**) Averaged expression of all genes in each group across three cell type classes. *$p<$ 0.05, **$p<$ 0.01, Wilcoxon signed rank sum test with Benjamini-Hochberg post hoc. Single-nucleus RNA-seq data of each cell type are from Allen BrainMap.

Hierarchical clustering analysis of epilepsy-associated genes across 49 cell clusters revealed a clear separation into three distinct groups: glutamatergic neurons, GABAergic interneurons, and non-neuronal cells (Fig.  6A, Supplemental Fig. 8). While most epilepsy genes showed relatively similar expression between GABAergic and glutamatergic neurons, some genes such as *GAD1* and *SLC6A1* that are cell type-specific markers, were significantly different between these two neuronal cell types as expected (Supplemental Fig. 3). Lastly, our analysis revealed that the average expression of DEEGs was significantly higher than the other two groups in GABAergic neurons, suggesting a critical role for this neuronal subtype in DEEG-associated epilepsy (Fig.  6B,C).

## Discussion

Here we systematically examined the expression pattern of 230 epilepsy-associated genes in human tissues, across development, and within brain-specific cell types to explore the relationship between gene expression and phenotypic manifestation.

Epilepsy is a common neurological disorder characterized by phenotypic diversity with a substantial genetic component to its etiology. Based on the clinical phenotypes curated in OMIM, we grouped epilepsy-associated genes into CEG, DEEG, and SRG. Our analysis demonstrates that CEGs and DEEGs are enriched in the adult CNS, DEEGs have the highest and most diversed temporal expression in the brain across development, and DEEGs have the highest expression in adult cortical GABAergic neurons relative to the other two groups of genes. The experssion patterns of genes within these groups indicate their important physiological roles within respective cell types, tissues, and developmental stages which, when disrupted by disease-associated mutations, likely drive the pathology and comorbidities associated with these conditions.

Epilepsy has been postulated to arise as a result of a network imbalance^12^, involving both excitatory and inhibitory neurons. Our cell type analyses demonstrate that most epilepsy-associated genes are similarly expressed in both types of neurons, at least in cortical brain regions. At the same time, DEEGs as a group are significantly more abundant in interneurons, suggesting a potentially strong contribution of inhibitory signaling in these epilepsy cases.

Importantly, we found that some genes are associated with divergent clinical phenotypes. For example, *SCN1A*, *SCN2A*, *SCN3A*, *SCN8A*, *SCN1B*, *SLC12A5*, *TBC1D24*, *GABRA1*, *GABRB3*, *GABRG2*, *HCN1*, *KCNQ2*, and *KCNT1* are shared between the CEG and DEEG groups. The fact that they are involved in two phenotypic outcomes suggests that the gene expression pattern cannot be the only source of phenotypic diversity. As a matter of fact, many studies have demonstrated that several factors can contribute to phenotypic diversity. First, the functional effect of the mutation on the encoded protein matters, which is especially true for ion channels. One prominent example is the *SCN1A* gene, which encodes voltage-gated sodium channel alpha subunit 1 (Nav1.1). While loss-of-function (LOF) variants in the *SCN1A* gene cause Dravet syndrome and genetic epilepsy with febrile seizures plus, gain-of-function (GOF) variants are associated with familial hemiplegic migraine^13–15^. Mechanistically, LOF and GOF *SCN1A* mutations can cause enhanced or diminished Nav1.1 ion channel activity, presumably leading to differential network excitability effects depending on the affected neuronal cell type. Consistently, many studies have reported that LOF of *SCN1A* predominantly affects GABAergic interneurons^16–25^, although other studies show that glutamatergic neurons are also affected^26–28^. Lastly, different variants may have different functional effects via altering splicing. Thompson and colleagues recently showed that different *SCN2A* variants exert different functional effects on neonatal versus adult *SCN2A* isoforms, leading to complex phenotypic outcomes in immature versus mature neurons^29^.

Second, diverse phenotypes could also arise as a result of gene-gene interactions. Several studies in *Drosophila* have demonstrated the contribution of gene-gene interactions to phenotypic diversity^30–33^. For example, using *Drosophila* mutants Ganetzky and Wu showed that one particular mutant with deficits in generating action potentials can suppress the behavioral defect caused by mutations at seven loci^30^. The contribution of gene-gene interactions to phenotypic diversity in neuronal excitability has also been shown in mouse models^34–37^. In these studies, mouse models carrying the gene and mutation of interest are generated and two mouse lines are then crossed to examine the enhancement or suppression of seizures caused by mutations in one gene. Using this approach, Kearney and Noebels’s groups examined genetic interactions between several ion channel gene pairs, including *CACNA1G* and *SCN2A/SCN1A*^34,35^, *KCNQ2* and *SCN2A*^36^, and *CACNA1A* and *KCNA1*^37^. In contrast to the extensive studies on the interaction of two ion channel genes, gene-gene interactions between an ion channel gene and a non-ion channel gene are less explored. A recent study by Seiffert *et al.* using cellular models demonstrated that different splicing isoforms or variants in a gene encoding fibroblast growth factor 12 (FGF12) affect the kinetics of Nav1.2 or Nav1.6, two ion channels encoded by *SCN2A* and *SCN8A*, respectively^38^.

Third, gene-environment interactions can also contribute to phenotypic variation. Environmental factors such as diet can affect phenotypic presentation. One prominent example is using the ketogenic diet for seizure control^39^. Notably, only some individuals respond well to the ketogenic diet (i.e., seizures are well controlled by the diet). It is now known that genetic factors underlie the differential responses^40^. In recent years, natural product-derived therapies have gained more attention for seizure control since the U.S. Food and Drug Administration (FDA) proved cannabidiol (CBD) for treating Dravet syndrome in 2018^41,42^. Mechanistically, CBD or other natural products act on specific biological components such as ion channels^42–44^. For instance, CBD has been shown to activate KCNQ channels^43^ and physically bind to SCN9A channels^44^. Gene-diet interactions are presumably more prominent for metabolic epilepsy genes. In these cases, genetic mutations often lead to compromised enzymatic activities, which diminishes or abolishes the conversion from substrates to products^45^. Dietary treatment can compensate for the deficiency, which has been demonstrated in many metabolic epilepsies, including for pyridoxine 5’-phosphate oxidase (PNPO) deficiency-induced epilepsy^46,47^. PNPO is a rate-limiting enzyme in vitamin B6 synthesis. Seizures in patients with severe PNPO deficiency responded well to pyridoxal 5’-phosphate (PLP), the product of PNPO. Our recent work in *Drosophila* carrying human mutant *PNPO* alleles demonstrates a specific allele-diet interaction in seizure and development in flies^48^.

Lastly, genetic mosaicism has been increasingly recognized to contribute to phenotypic diversity in human diseases, including epilepsy^49,50^. Genetic mosaicism refers to the accumulation of somatic mutations in the genome that arise during the developing stages after gametogenesis. Due to the stochasticity of the event, different types of cells can be affected, ultimately leading to a spectrum of corresponding phenotypes. Thus, while collectively there is a correlation between gene expression and phenotypic presentation, each gene itself also encompasses additional complexity due to the functional effects of the mutation on the protein, gene-gene, and gene-environment interactions as well as genetic mosaicism.

There has been tremendous progress in epilepsy genetics in the past three decades^4,51^. Recent efforts have been put into curating epilepsy genes^5,6,11,52^, but less so for associated phenotypes. Given the complexity of the clinical phenotypes associated with epilepsy and the lack of standardized descriptions across different studies, here we relied solely on the phenotypic description curated in OMIM, a suboptimal database for epilepsy studies due to the rapid development of the field. As the epilepsy gene and phenotype curation continuously evolves, a more complete and accurate correlation may be explored.

In summary, in this study we grouped and analyzed the expression of 230 epilepsy-associated genes at tissue, developmental stage, and single-cell levels. Our work provides an overview of the expression pattern of an inclusive collection of epilepsy-associated genes across tissues, developmental stages, and neural subtypes. The analyses and the established pipeline we present here will be a useful resource for the community, serving as a starting point for our understanding of gene expression patterns and phenotypic diversity in epilepsy.

## Methods

### Categorization of epilepsy-associated genes based on phenotypes

The genotype-phenotype data were obtained from OMIM (accessed in November, 2022)^10^. A total of 8533 items were curated in OMIM, including disorders with unknown genetic defects (n = 33), disorders with genes but no mutation identified (n = 1051), disorders with genes and mutations identified (n = 7285), and disorders with multiple genes involved through deletion or duplication (n = 164). We queried related items from the group of disorders with genes and mutations identified using the following terms: [E/e]pilepsy, [E/e]pilepsies, [E/e]pileptic, [S/s]eizure, and [S/s]eizures, which led to 282 items and 247 unique genes. These 247 genes were further cross-referenced with two recent studies by Macnee *et al.*^6^ and Oliver *et al.*^11^ to ensure their validity as epilepsy-associated genes (see Fig.  1).

### Functional classification of epilepsy-associated genes

Functional classification of epilepsy-associated genes was performed using the PANTHER classification system^53^. Based on the assigned PANTHER Protein Class, we grouped genes with known functions into one of the following seven categories: enzyme, ion channel, transporter/receptor, signaling, transcription/translation, cytoskeleton, and extracellular matrix. Genes with unknown functions were grouped into the category of ‘unknown’.

### Expression of epilepsy-associated genes in human tissues

Tissue-specific transcriptomes were obtained from the Genotype-Tissue Expression (GTEx) project (Open access data, V8 dbGaP Accession phs000424.v8.p2)^7^. This dataset consists of bulk RNA sequencing data from 663 donors across 52 tissues as well as cultured fibroblast cells and transformed lymphocytes. For the clustering analyses we considered the mean expression for each gene across 52 tissues. The final data included 230 epilepsy-associated genes.

### Expression of epilepsy-associated genes in the brain across development

Developmental transcriptomes in the brain were obtained from BrainSpan (RNA-Seq Gencode v10 summarized to genes)^9^. This dataset consists of bulk RNA sequencing data in 26 brain sub-regions in 11 developmental stages, from embryonic stages to adulthood, with the sample size of each region ranging from 229 to 8244 in total. We selected epilepsy-associated genes in this dataset and grouped every single stage into one of five periods: prenatal (4-37 post conception weeks), infancy (birth-18 months), childhood (19 months-11 years), adolescence (12–19 years), and adulthood (20–60+ years), based on the criteria used by BrainSpan^9^. We analyzed 198 epilepsy-associated genes that had expression data across all five developmental periods in 16 brain sub-regions. Brain regions with no data for all periods were excluded.

### Expression of epilepsy-associated genes in brain cell types

Brain cell-type specific transcriptomes were obtained from BrainMap^8^. This dataset consists of single-nucleus RNA sequencing data in 49,474 nuclei from 6 areas of the human cortex (primary motor cortex, primary sensory cortex, primary auditory cortex, primary visual cortex, middle temporal gyrus, and anterior cingulate gyrus). We selected individual cells that are molecularly defined (i.e., glutamatergic, GABAergic, or non-neuronal cells) and exhibit expression of epilepsy-associated genes. The final analyses include 205 epilepsy-associated genes in 49 clusters from 10,045 single nuclei.

### Supplementary Information

Supplementary Information.

Supplementary Figure 1.

Supplementary Figure 2.

Supplementary Figure 3.

Supplementary Figure 4.

Supplementary Figure 5.

Supplementary Figure 6.

Supplementary Figure 7.

Supplementary Figure 8.

## Data Availability

Analyses were performed in R (www.R-project.org). The Circos plot and heatmaps were generated using the Circlize^54^ and Complex Heatmap package^55^. The R packages and codes for data analysis can be found in https://github.com/Lilywindy2022/Epilepsy-gene-expression.
