# Supplementary figures and images for: Integrative analysis of epilepsy-associated genes reveals expression-phenotype correlations
Source: Sci Rep. 2024 Feb 13;14:3587. doi: 10.1038/s41598-024-53494-2 (PMC10864290; doi:10.1038/s41598-024-53494-2)

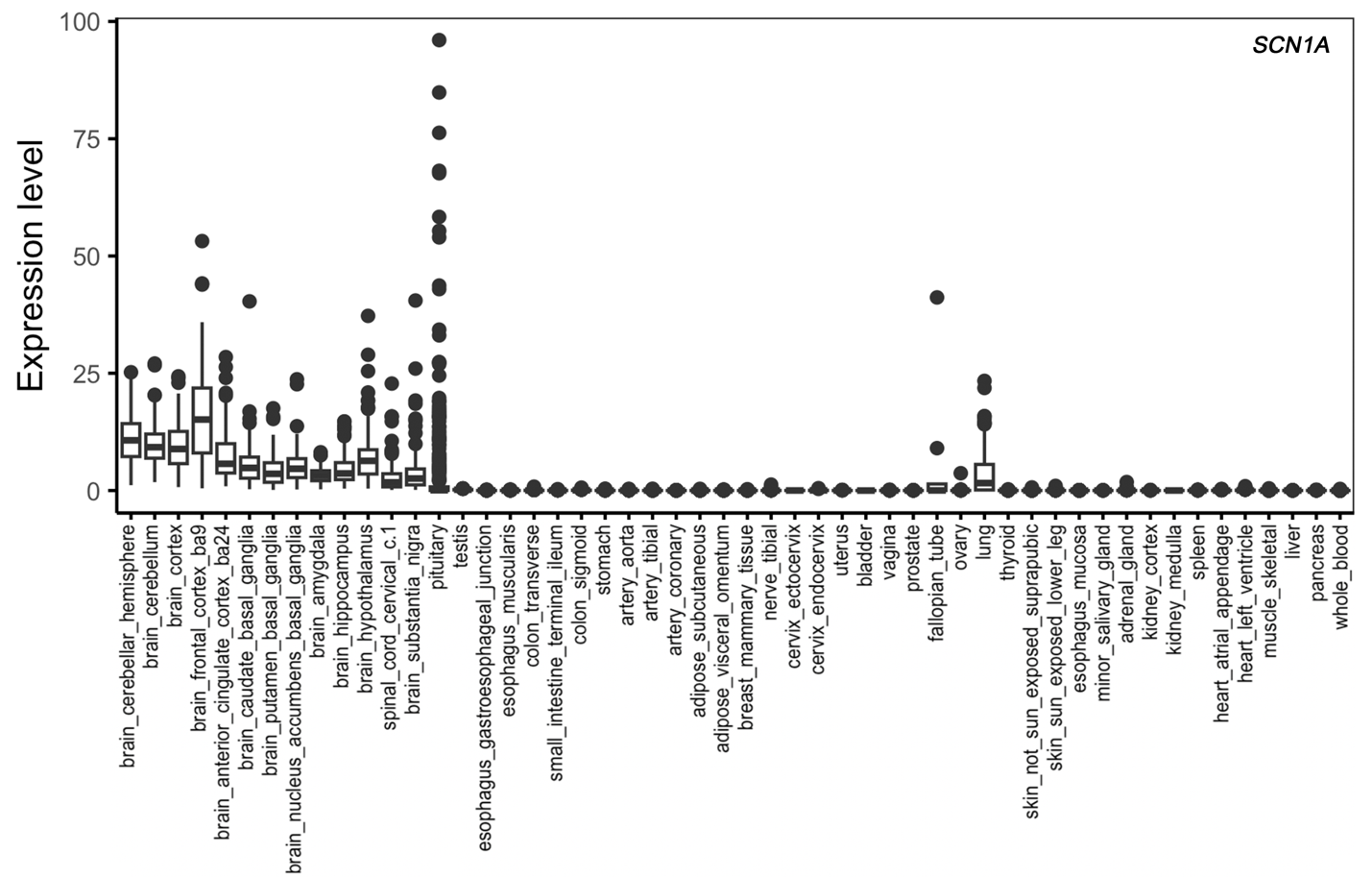


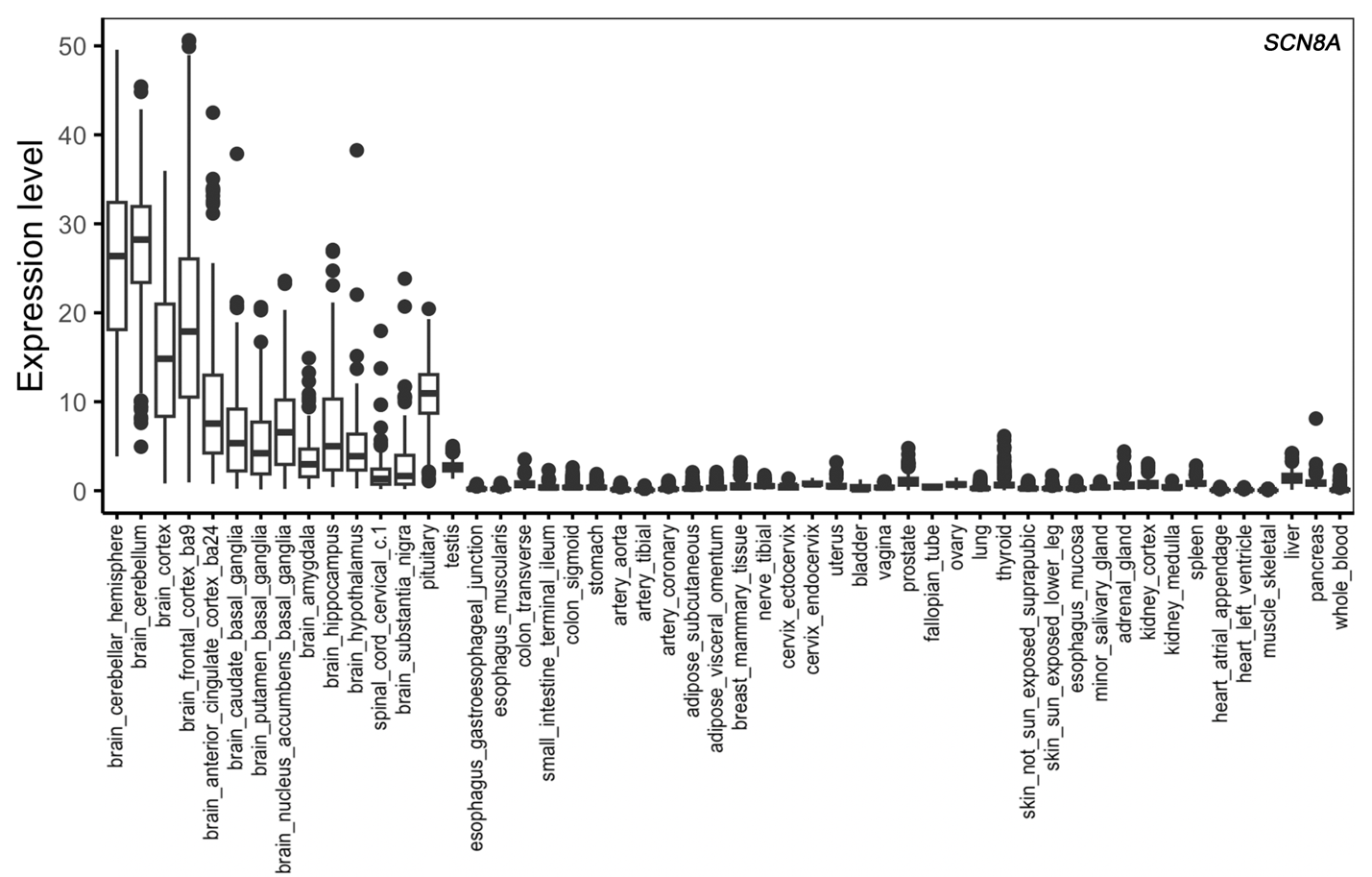


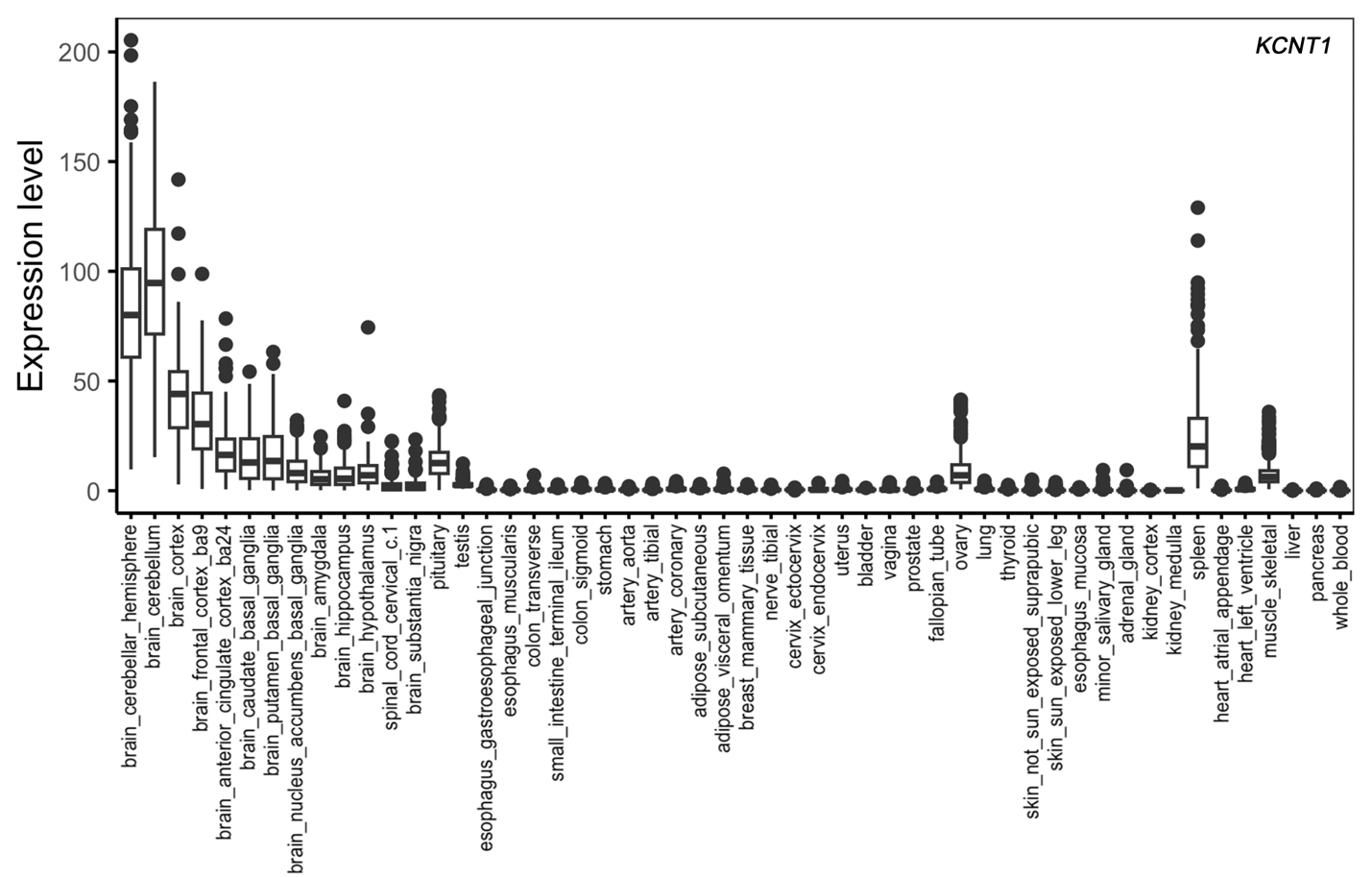


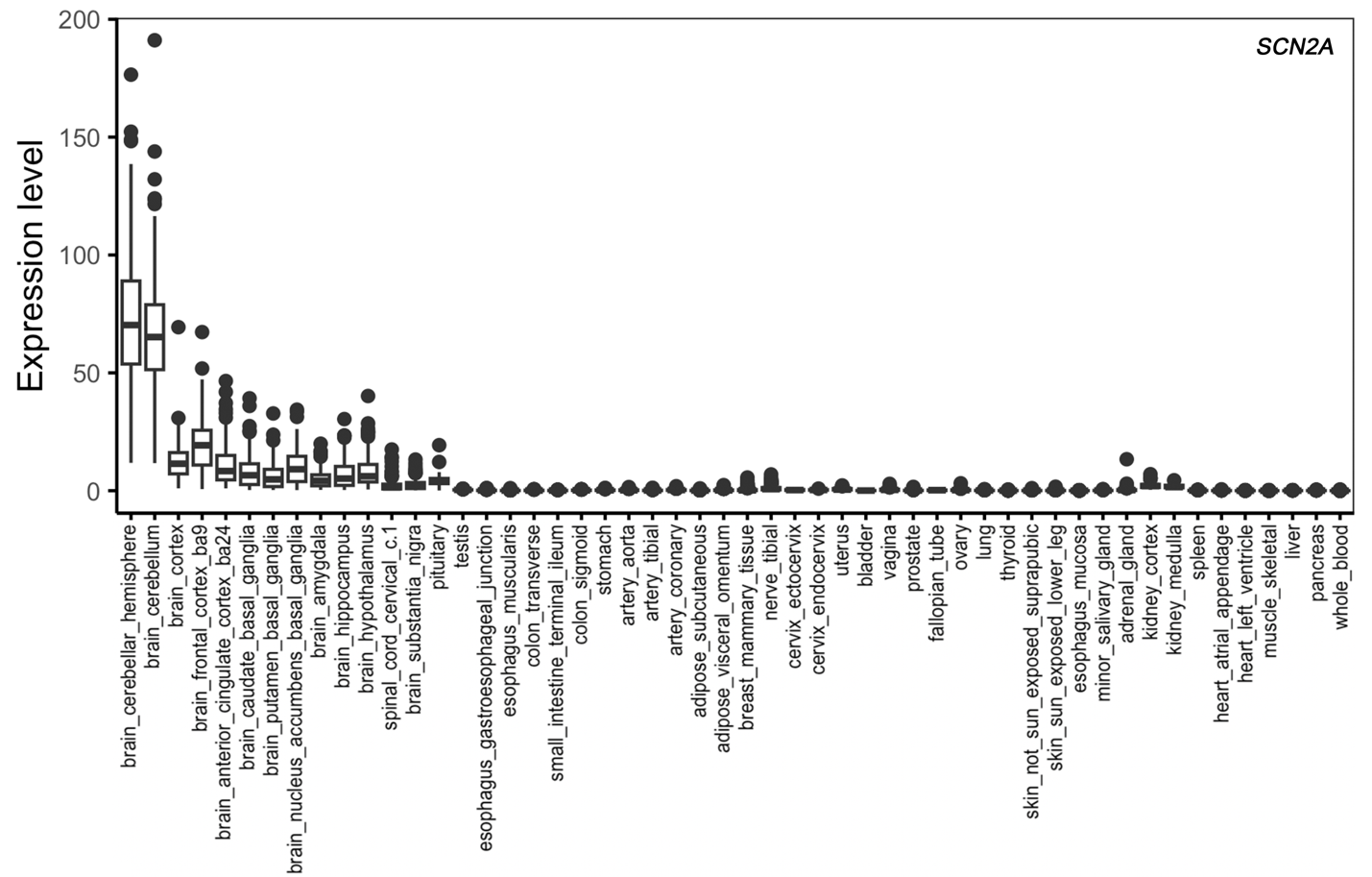


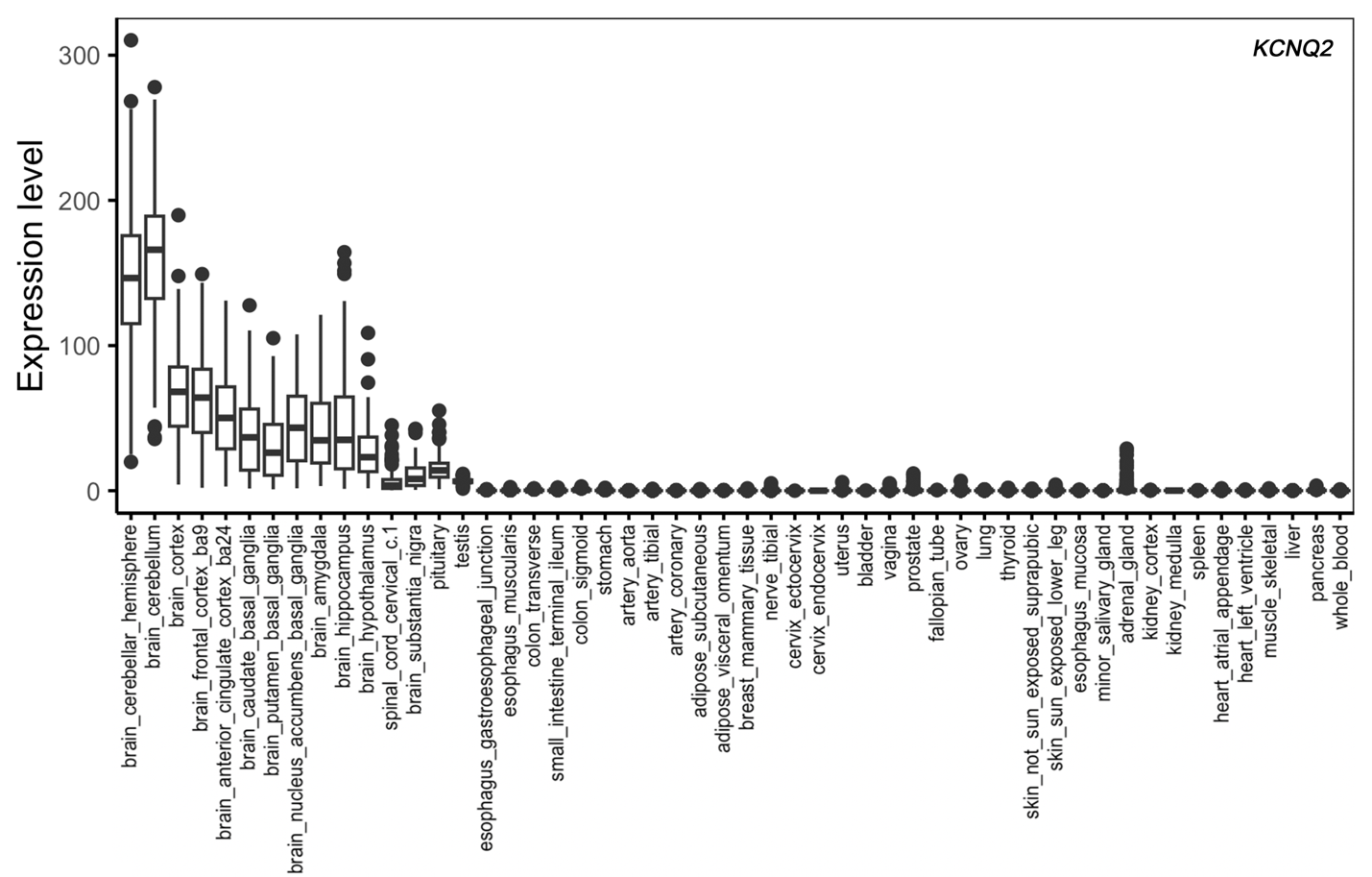


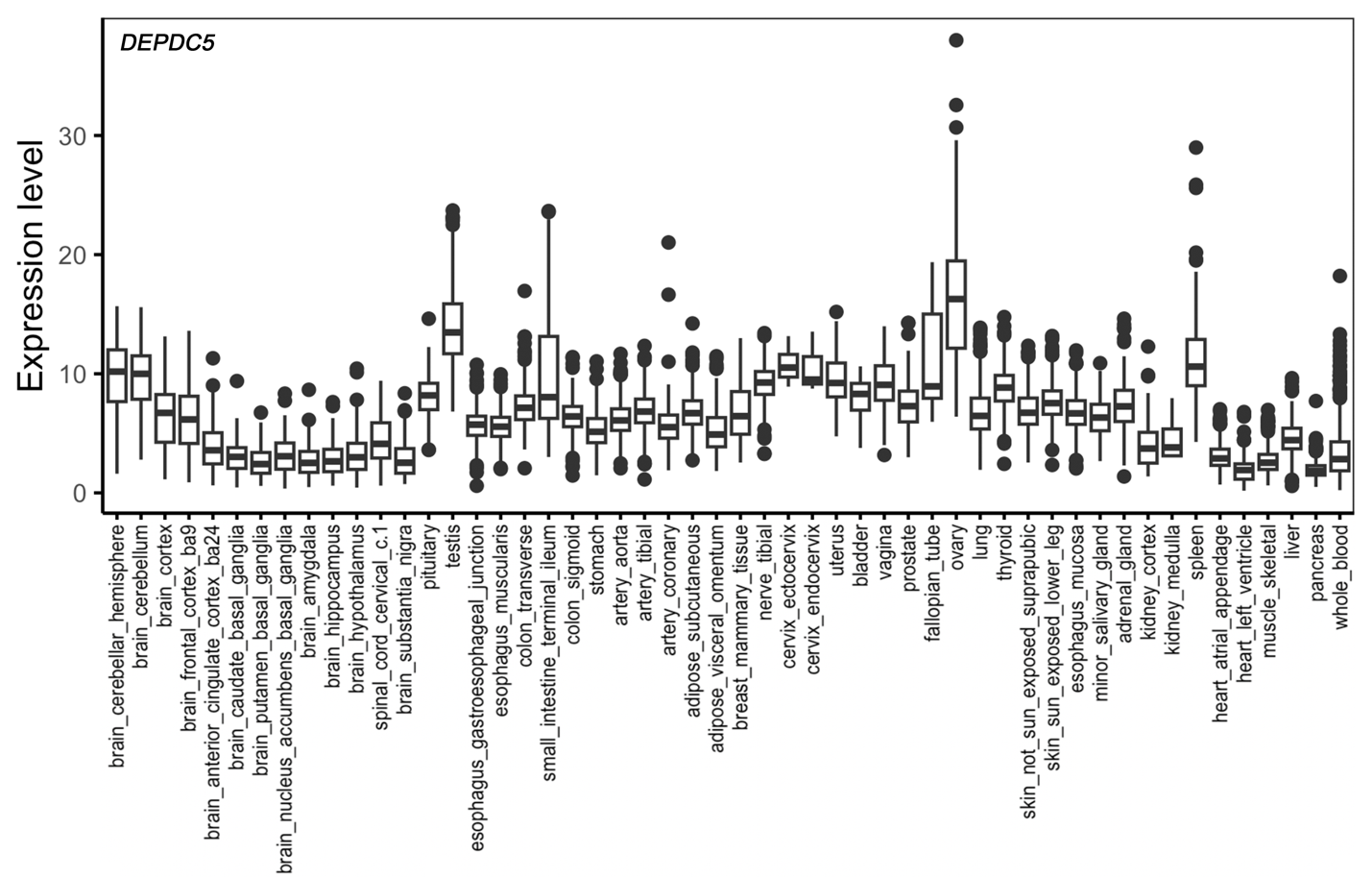


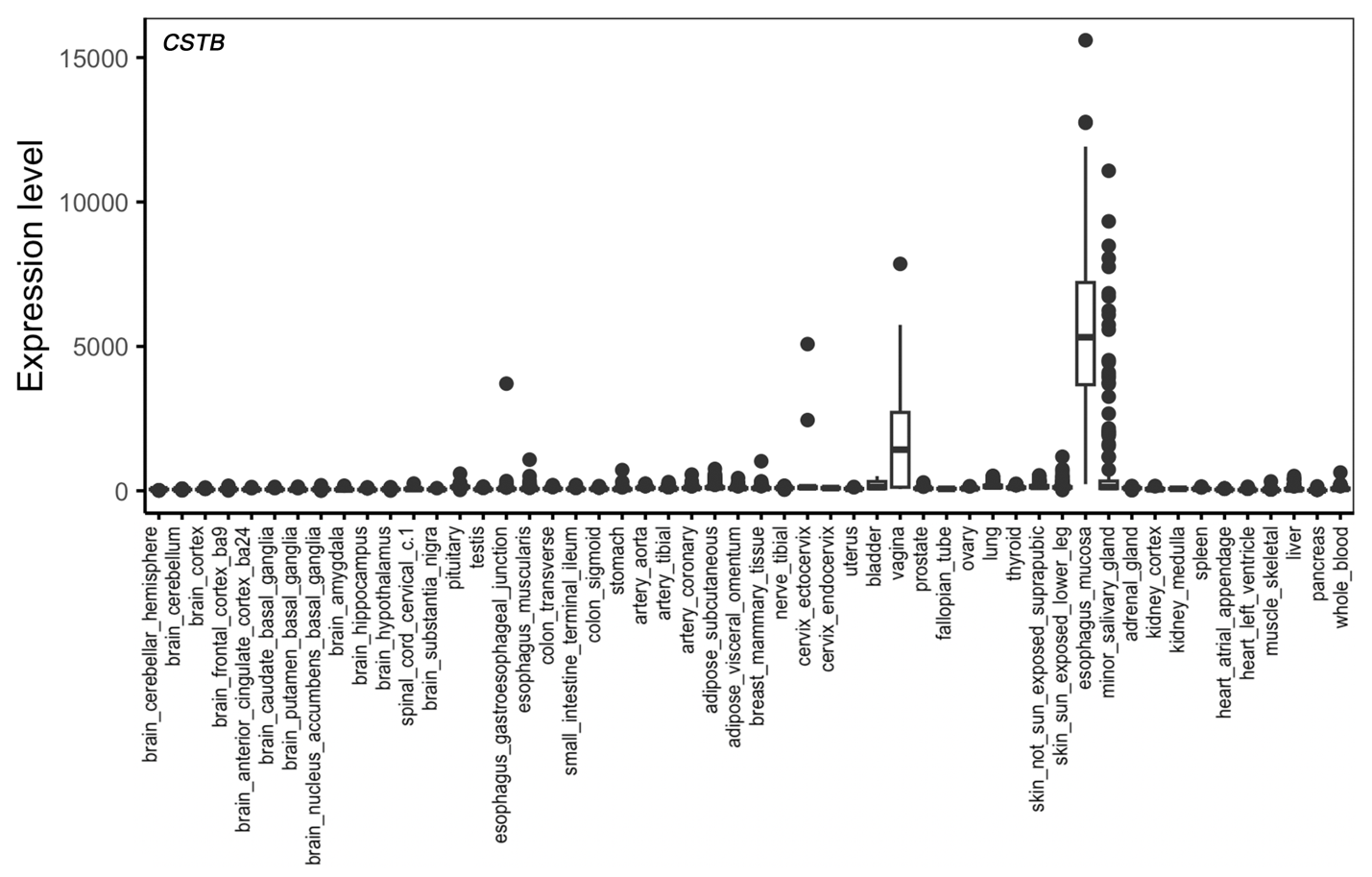


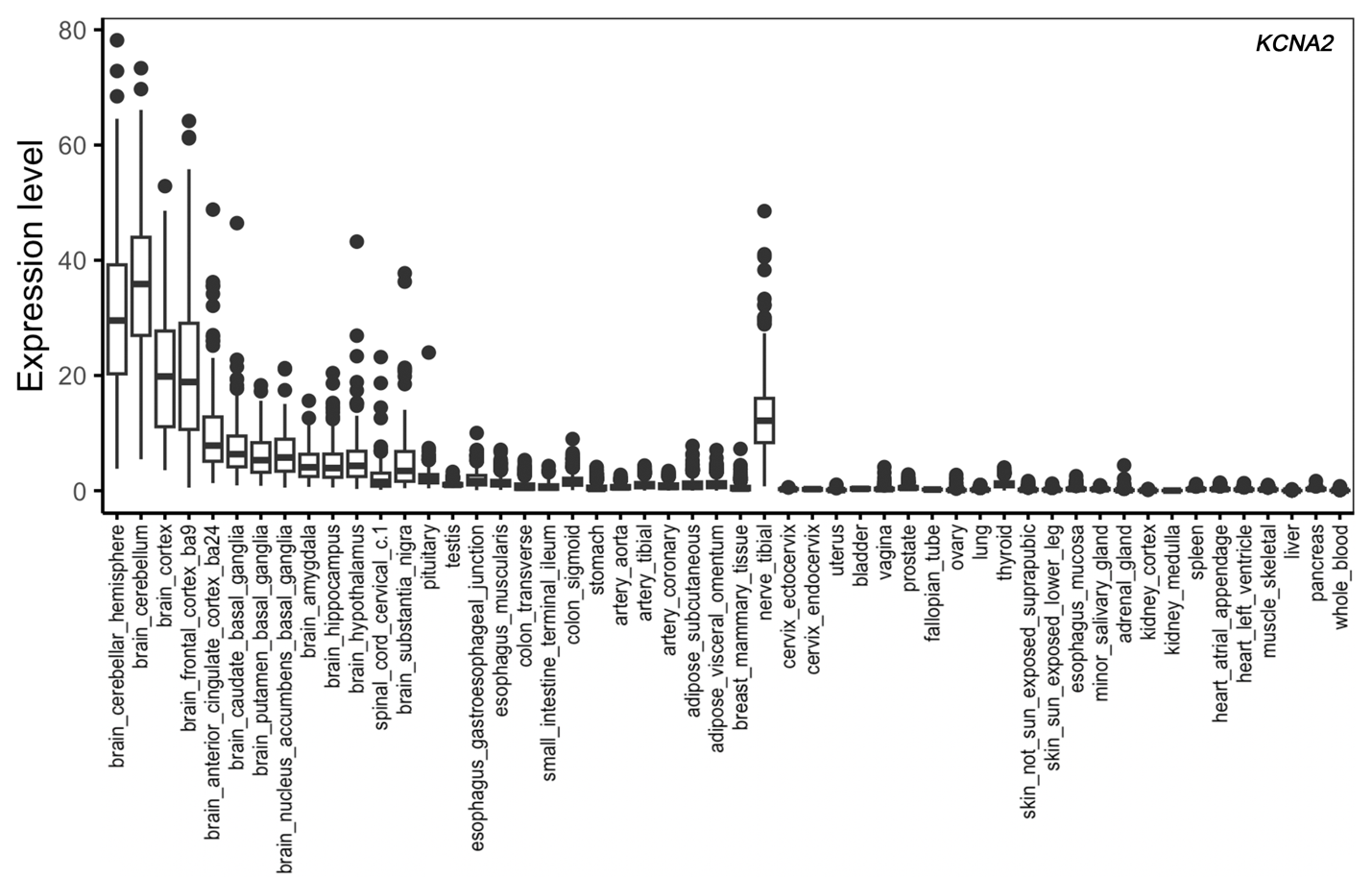


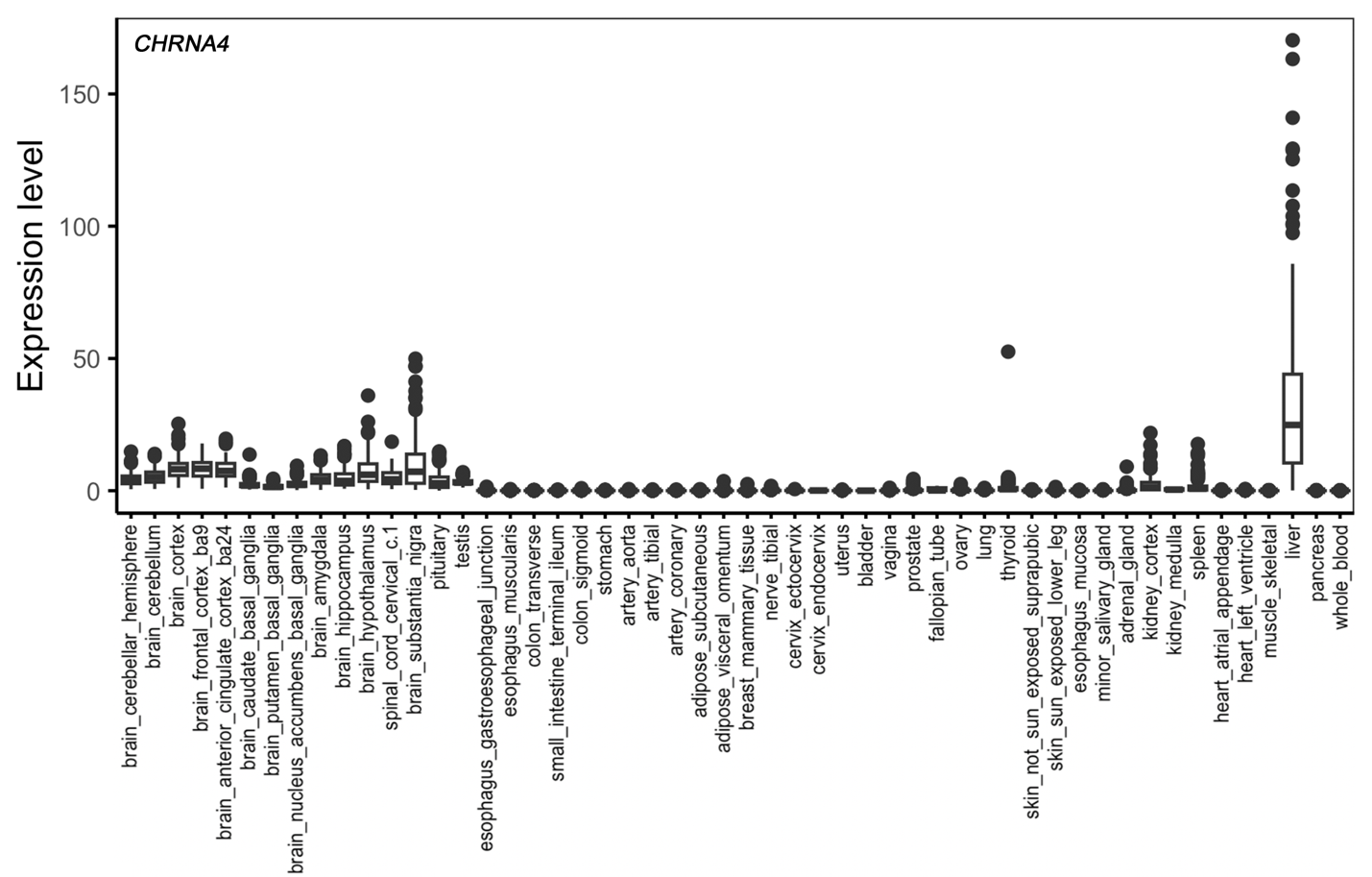


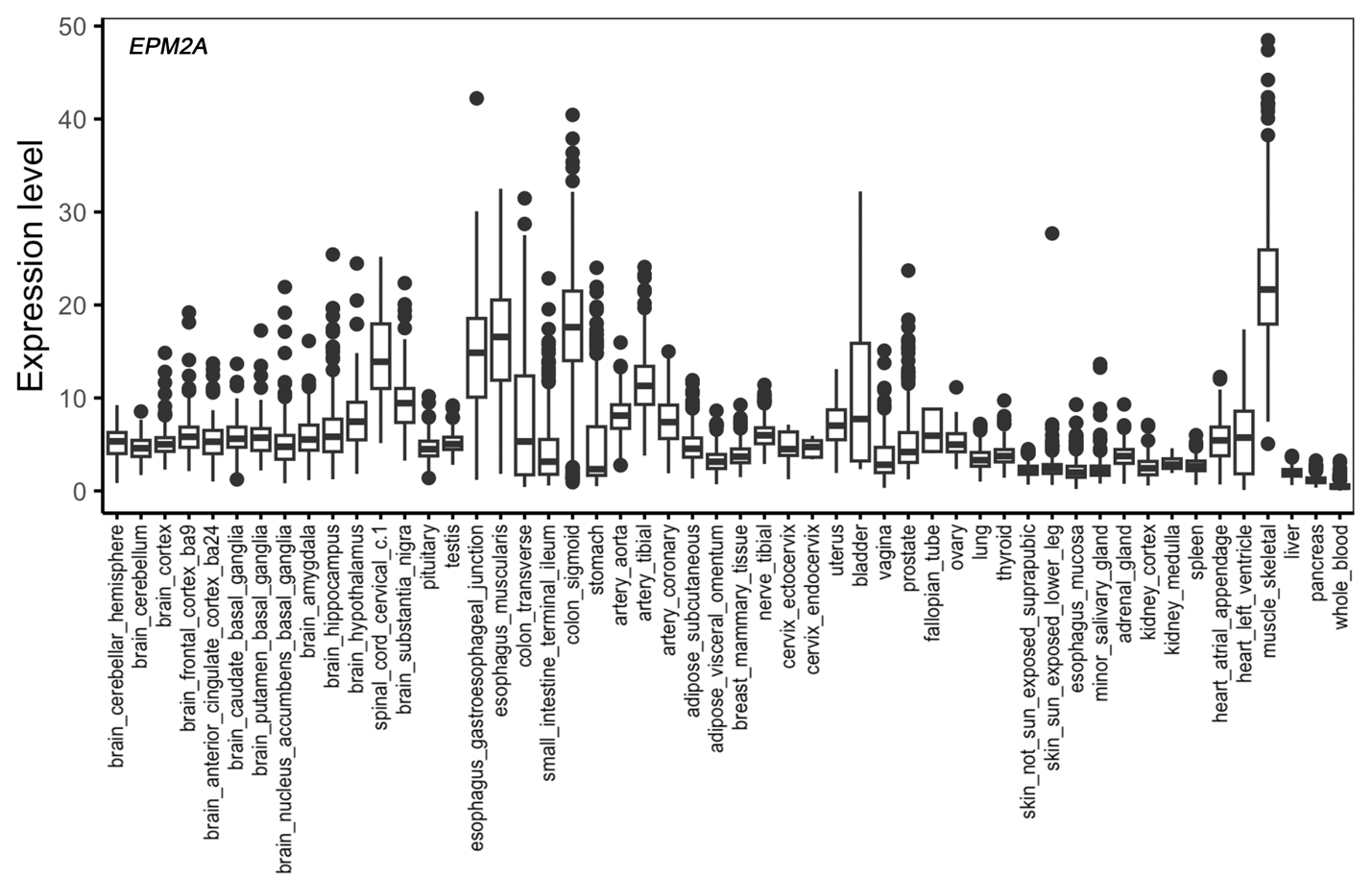


**Supplemental Figure 1. Expression of top 10 studied genes across human tissues.**

Supplement: Supplementary file 2 — Supplementary Figure 1. [file 41598_2024_53494_MOESM2_ESM.docx]
