# Supplementary material for: Integrative analysis of epilepsy-associated genes reveals expression-phenotype correlations
Source: Sci Rep. 2024 Feb 13;14:3587. doi: 10.1038/s41598-024-53494-2 (PMC10864290; doi:10.1038/s41598-024-53494-2)
Supplement: Supplementary file 3 — Supplementary Figure 2. [file 41598_2024_53494_MOESM3_ESM.docx]

**
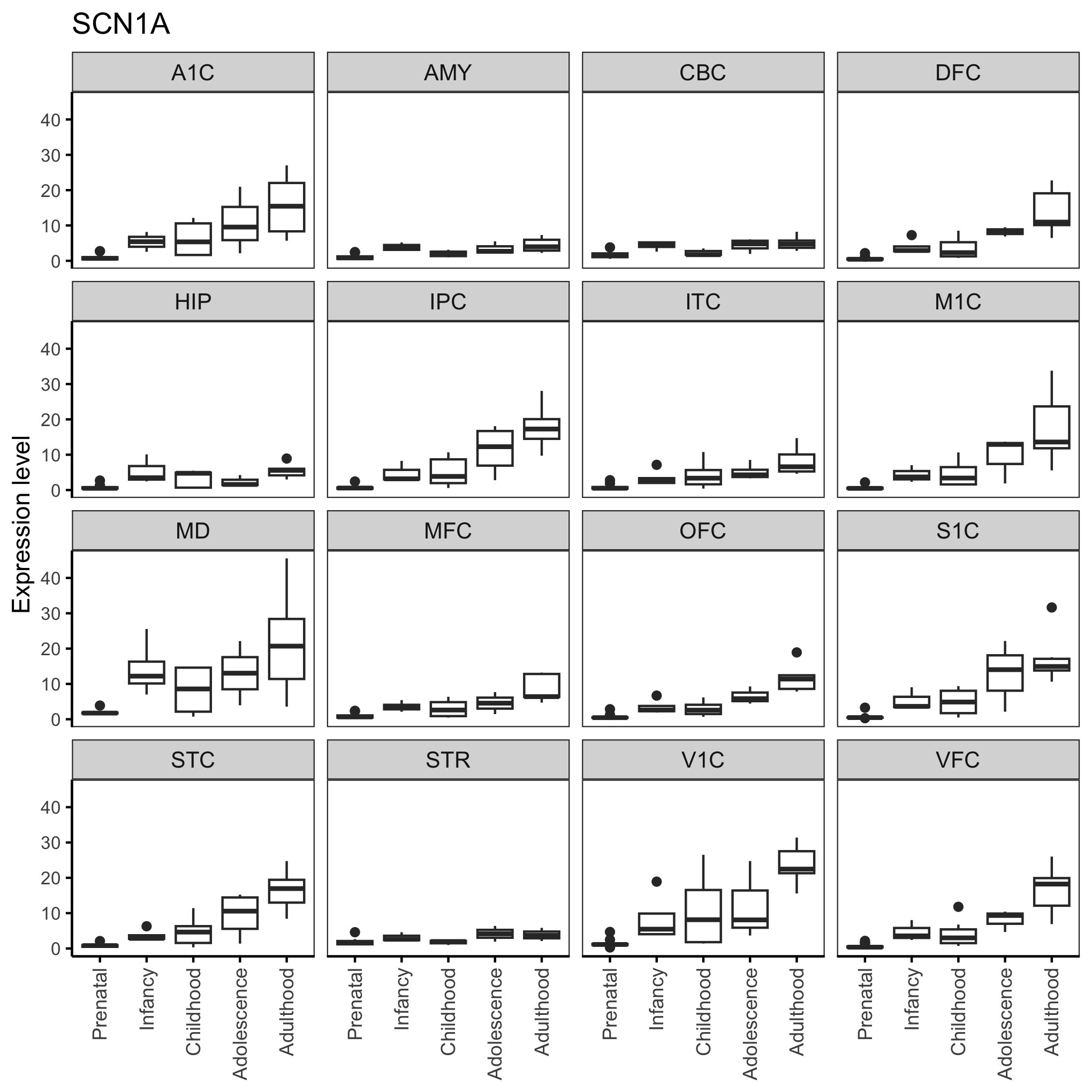
**

**
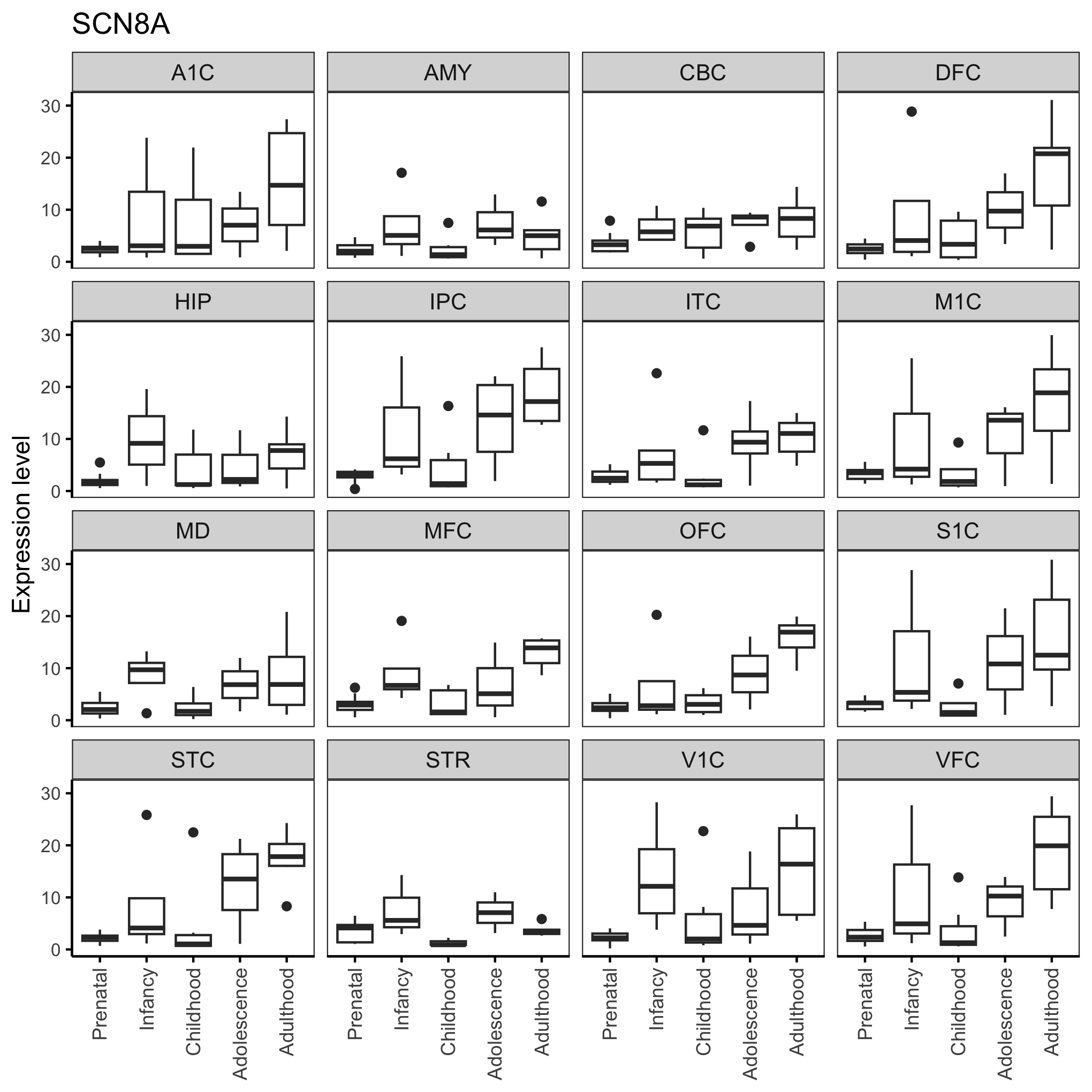
**

**
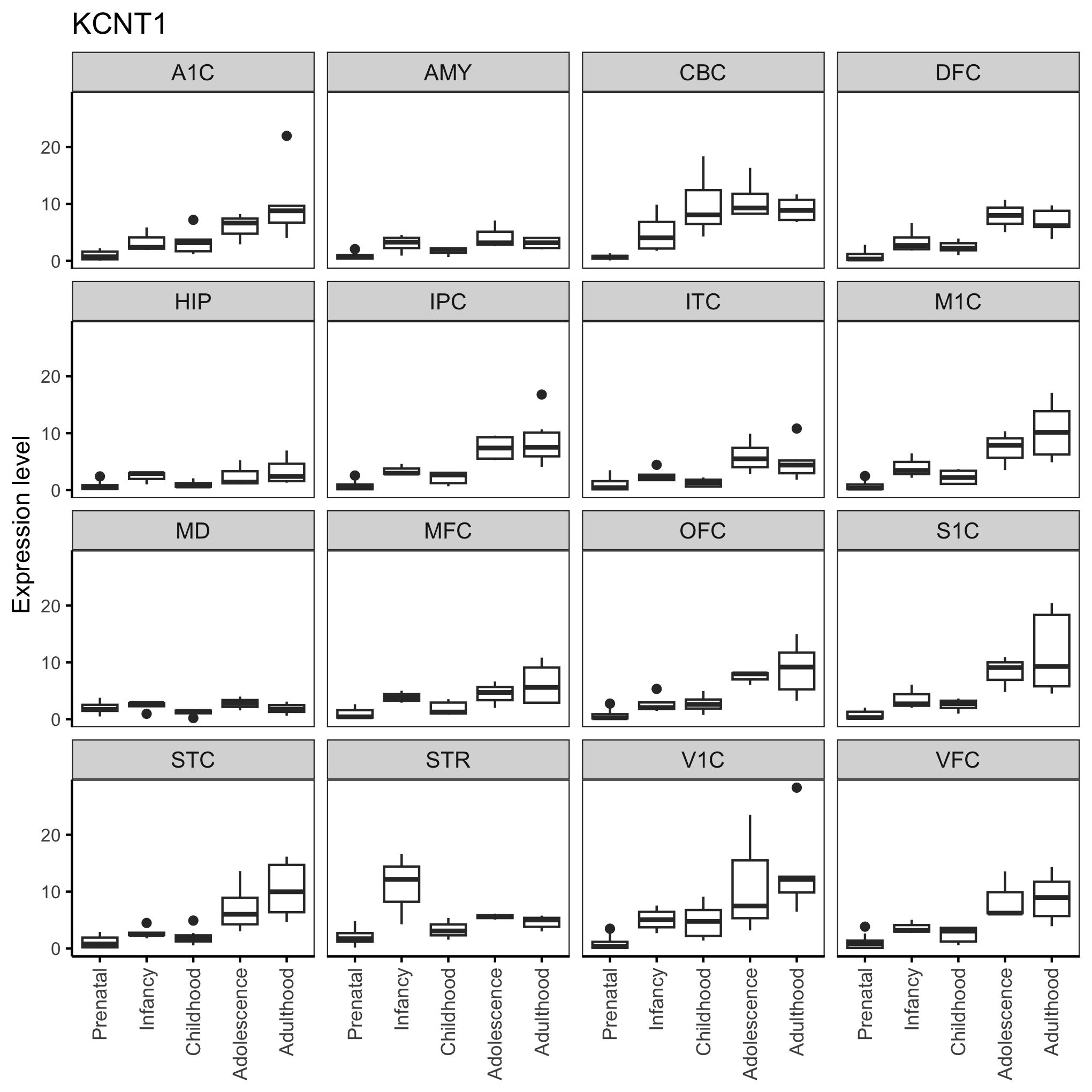
**

**
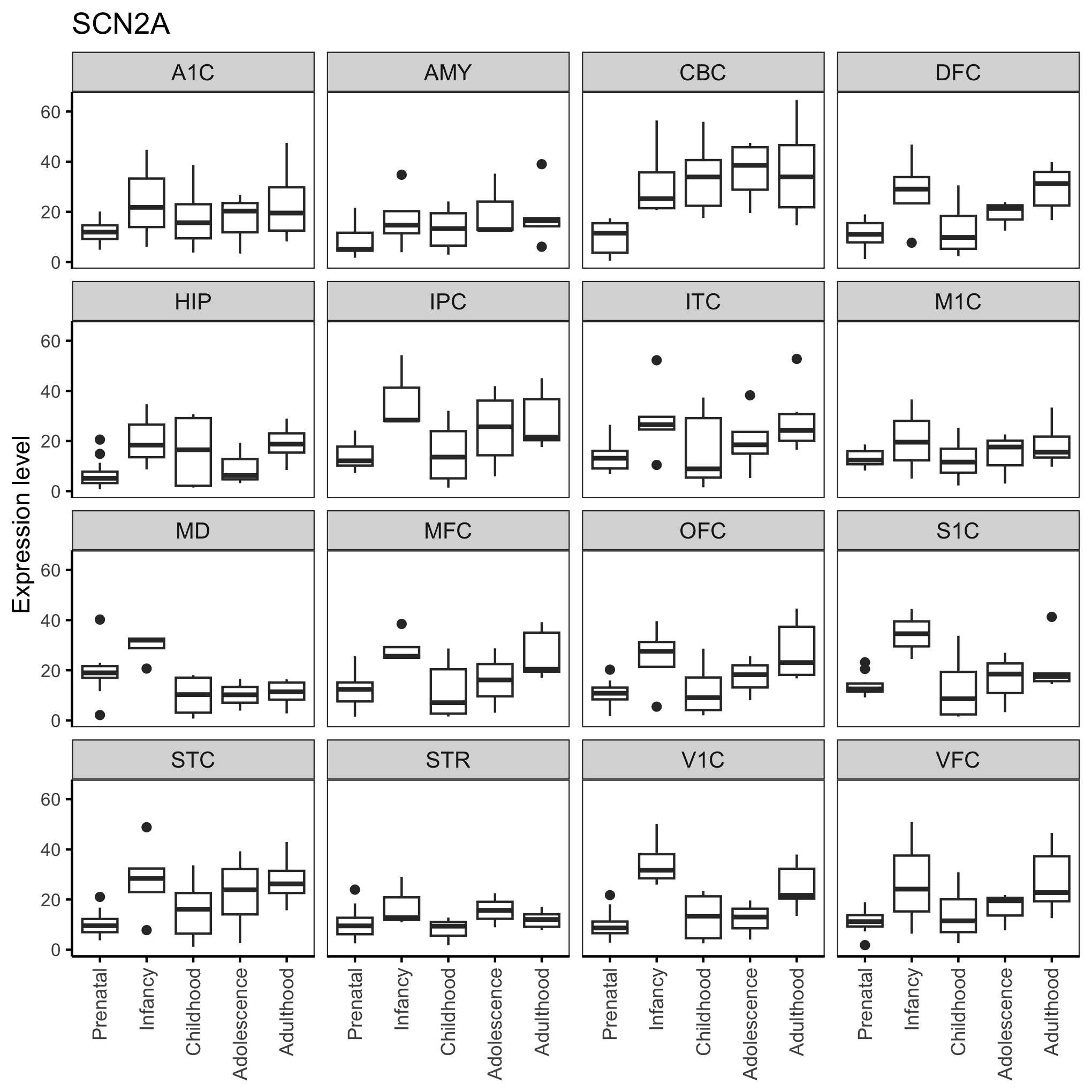
**

**
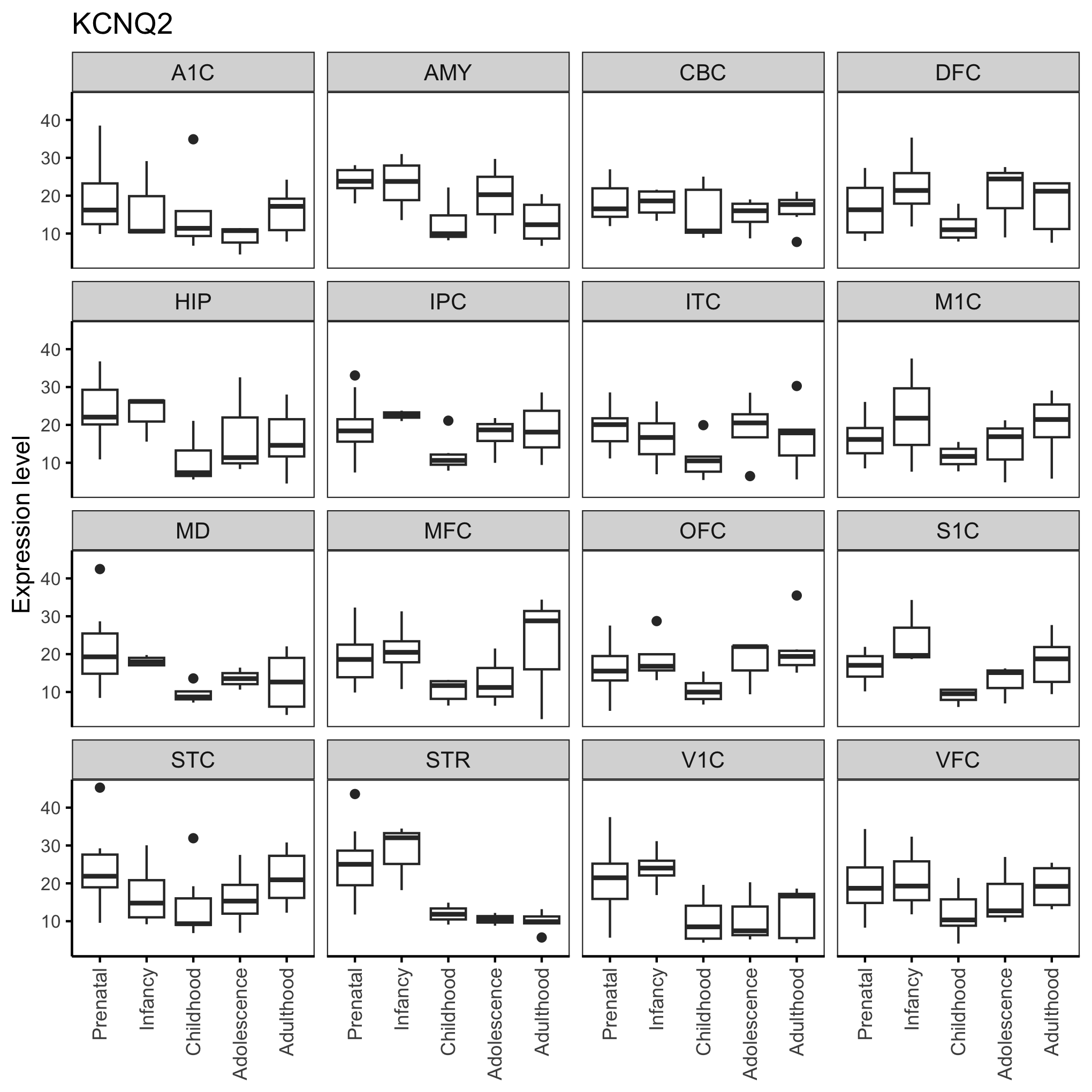
**

**
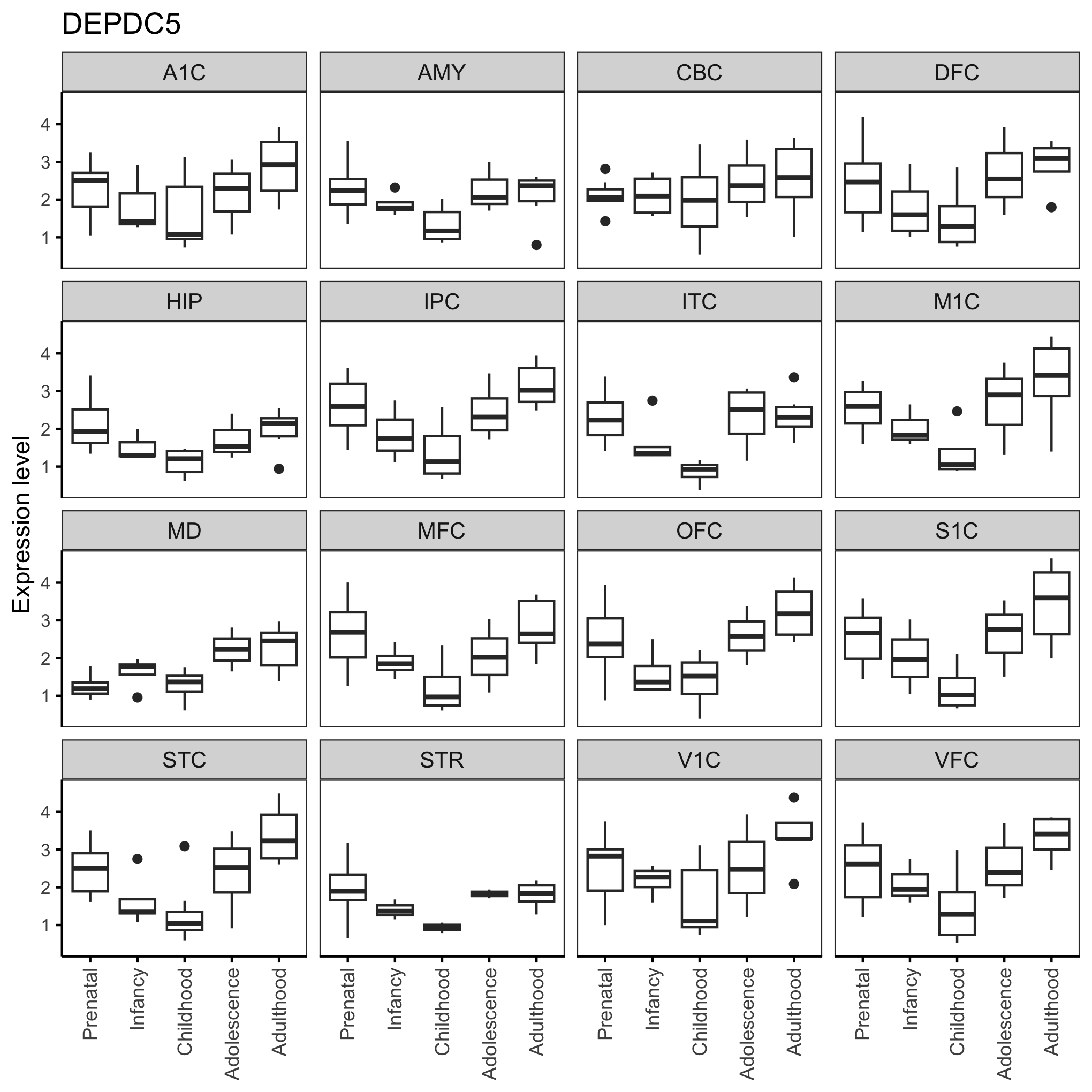
**

**
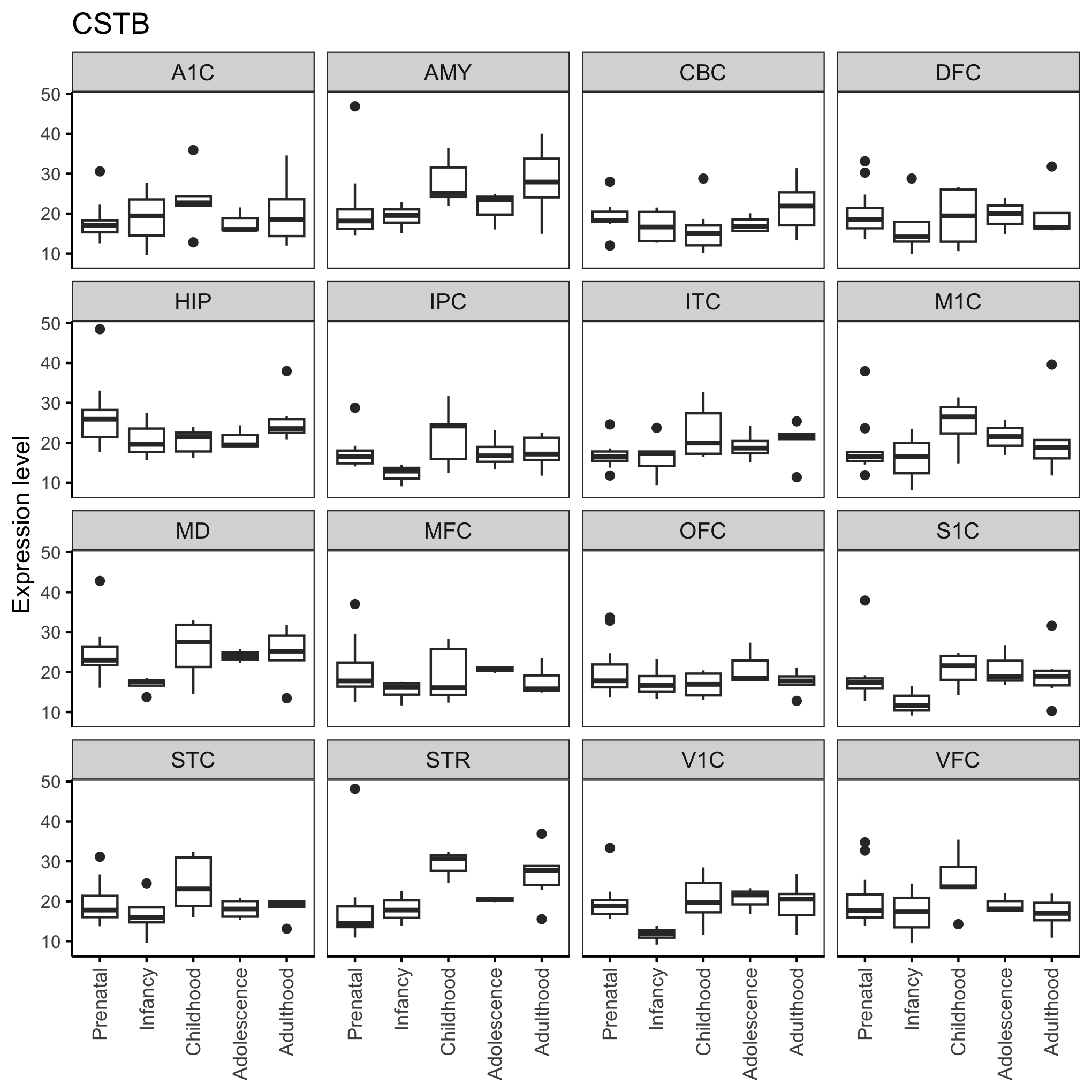
**

**
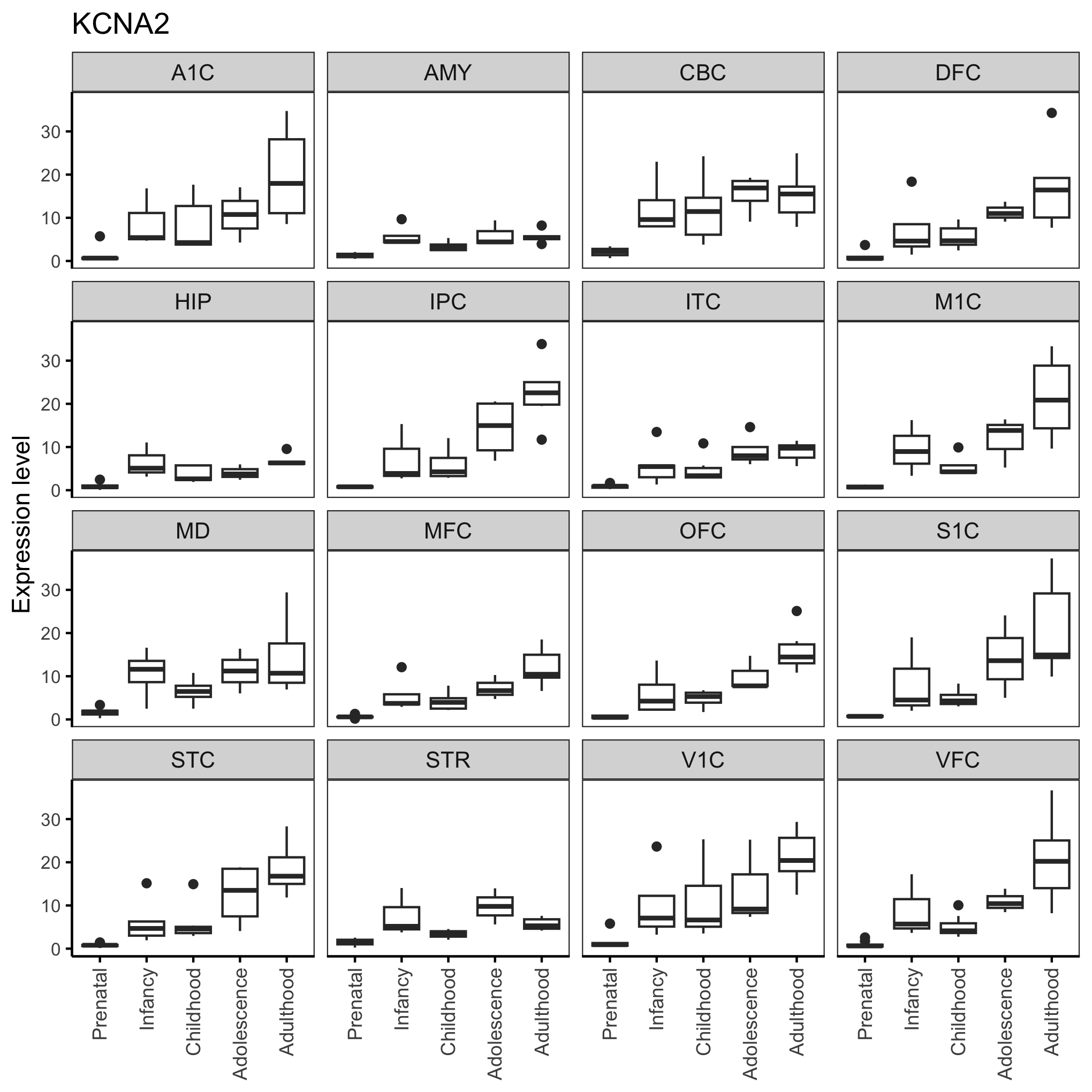
**

**
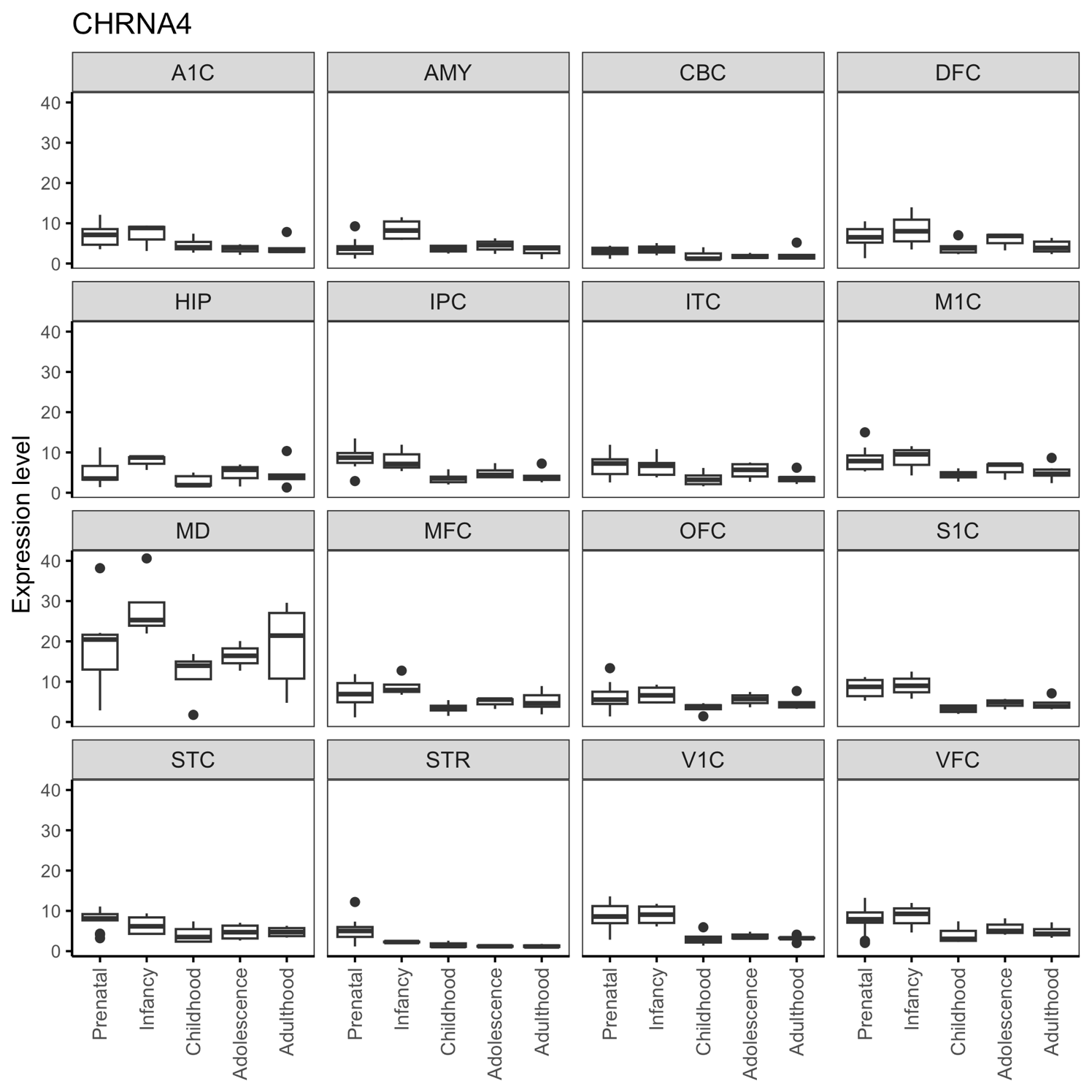
**

**
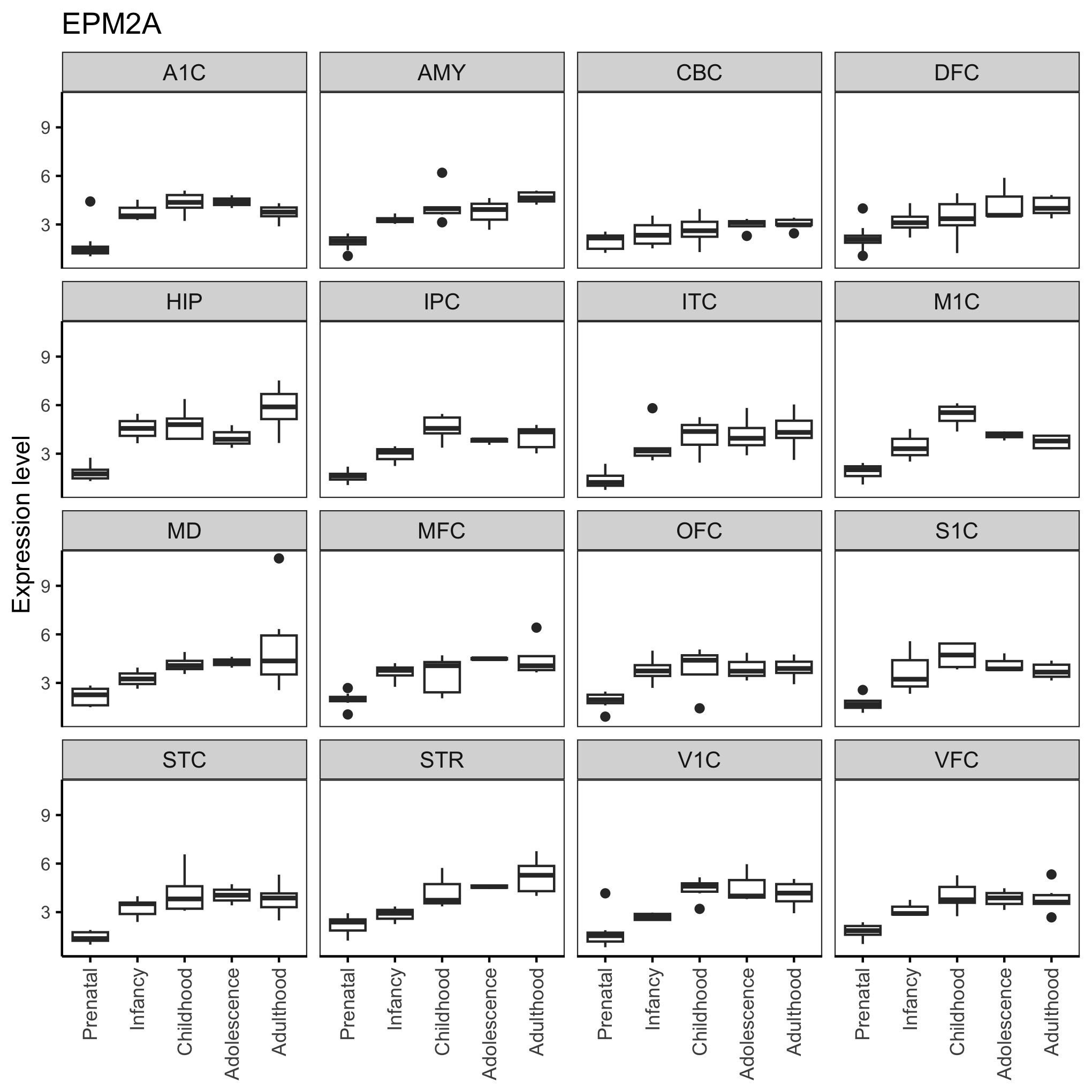
**

**Supplemental Figure 2. Expression of top 10 studied genes in different brain regions across development.**
