# Supplementary material for: Integrative analysis of epilepsy-associated genes reveals expression-phenotype correlations
Source: Sci Rep. 2024 Feb 13;14:3587. doi: 10.1038/s41598-024-53494-2 (PMC10864290; doi:10.1038/s41598-024-53494-2)
Supplement: Supplementary file 6 — Supplementary Figure 5. [file 41598_2024_53494_MOESM6_ESM.docx]

**
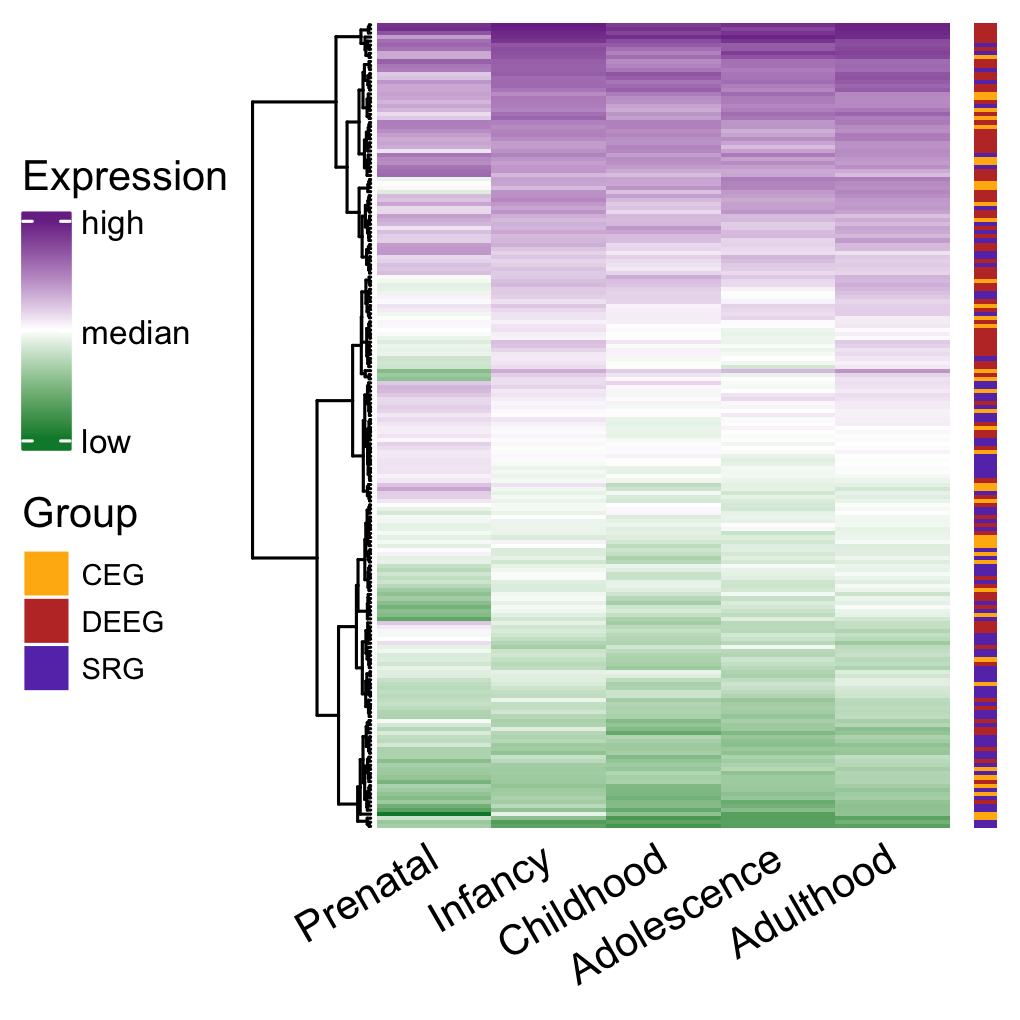
**

**Supplemental Figure 5. Expression of the three groups of epilepsy-associated genes in the hippocampus with hierarchical clustering across genes.**
