# Supplementary material for: Integrative analysis of epilepsy-associated genes reveals expression-phenotype correlations
Source: Sci Rep. 2024 Feb 13;14:3587. doi: 10.1038/s41598-024-53494-2 (PMC10864290; doi:10.1038/s41598-024-53494-2)
Supplement: Supplementary file 7 — Supplementary Figure 6. [file 41598_2024_53494_MOESM7_ESM.docx]

**
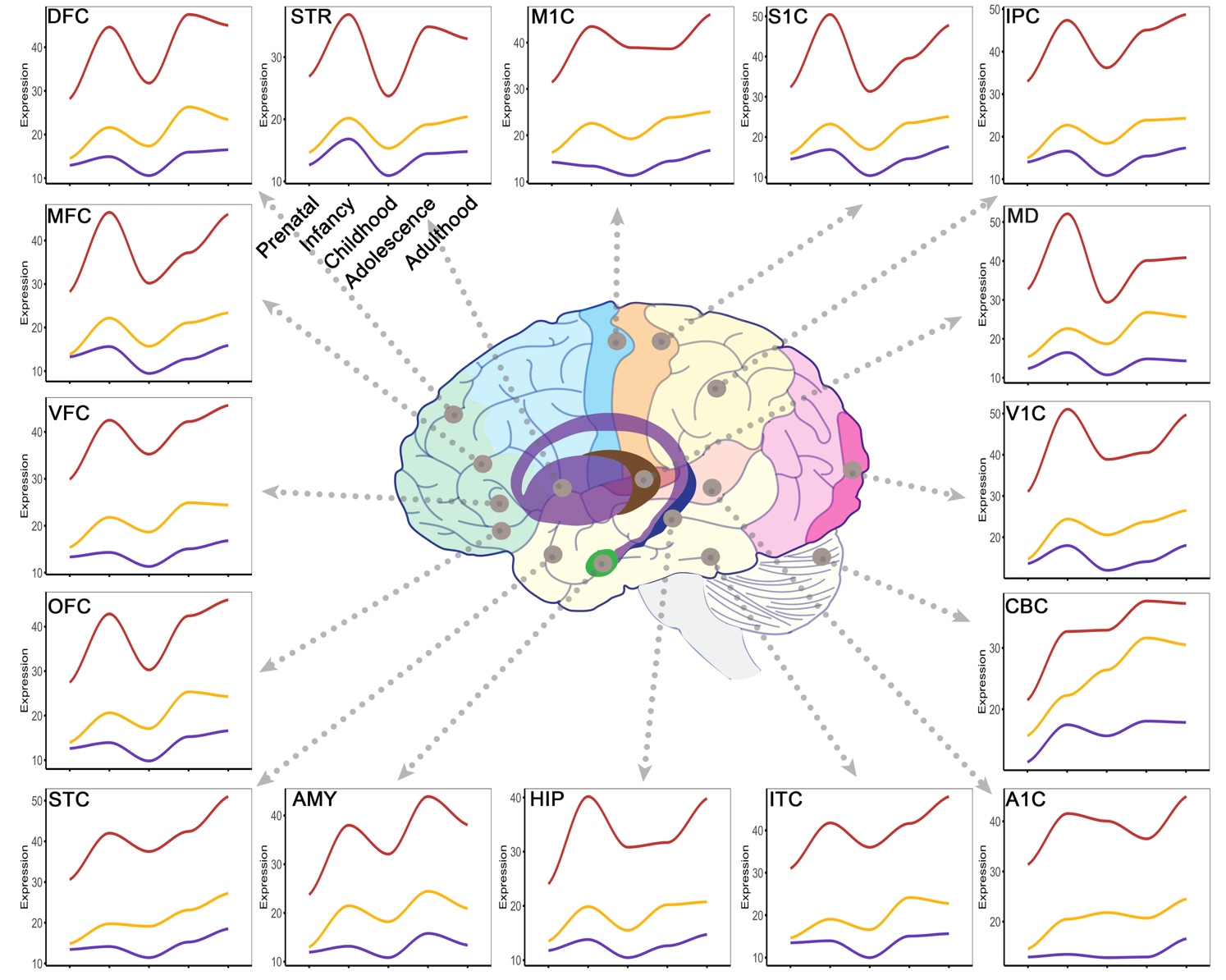
**

**Supplemental Figure 6. Averaged expression of epilepsy-associated genes within each group across development.** Red line: DEEG; orange: CEG; purple: SRG. Bulk RNA-seq data are from Allen BrainSpan. DFC: dorsolateral prefrontal cortex; MFC: anterior cingulate cortex; VFC: ventrolateral prefrontal cortex; OFC: orbital frontal cortex; STC: posterior superior temporal cortex; AMY: amygdala; HIP: hippocampus; ITC: inferolateral temporal cortex; A1C: primary auditory cortex; CBC: cerebellar cortex; V1C: primary visual cortex; MD: Mediodorsal nucleus of thalamus; IPC: posteroventral parietal cortex; S1C: primary somatosensory cortex; M1C: primary motor cortex; STR: striatum. See Supplemental Fig. 7 for significance levels.
