# Supplementary material for: Integrative analysis of epilepsy-associated genes reveals expression-phenotype correlations
Source: Sci Rep. 2024 Feb 13;14:3587. doi: 10.1038/s41598-024-53494-2 (PMC10864290; doi:10.1038/s41598-024-53494-2)
Supplement: Supplementary file 8 — Supplementary Figure 7. [file 41598_2024_53494_MOESM8_ESM.docx]

**
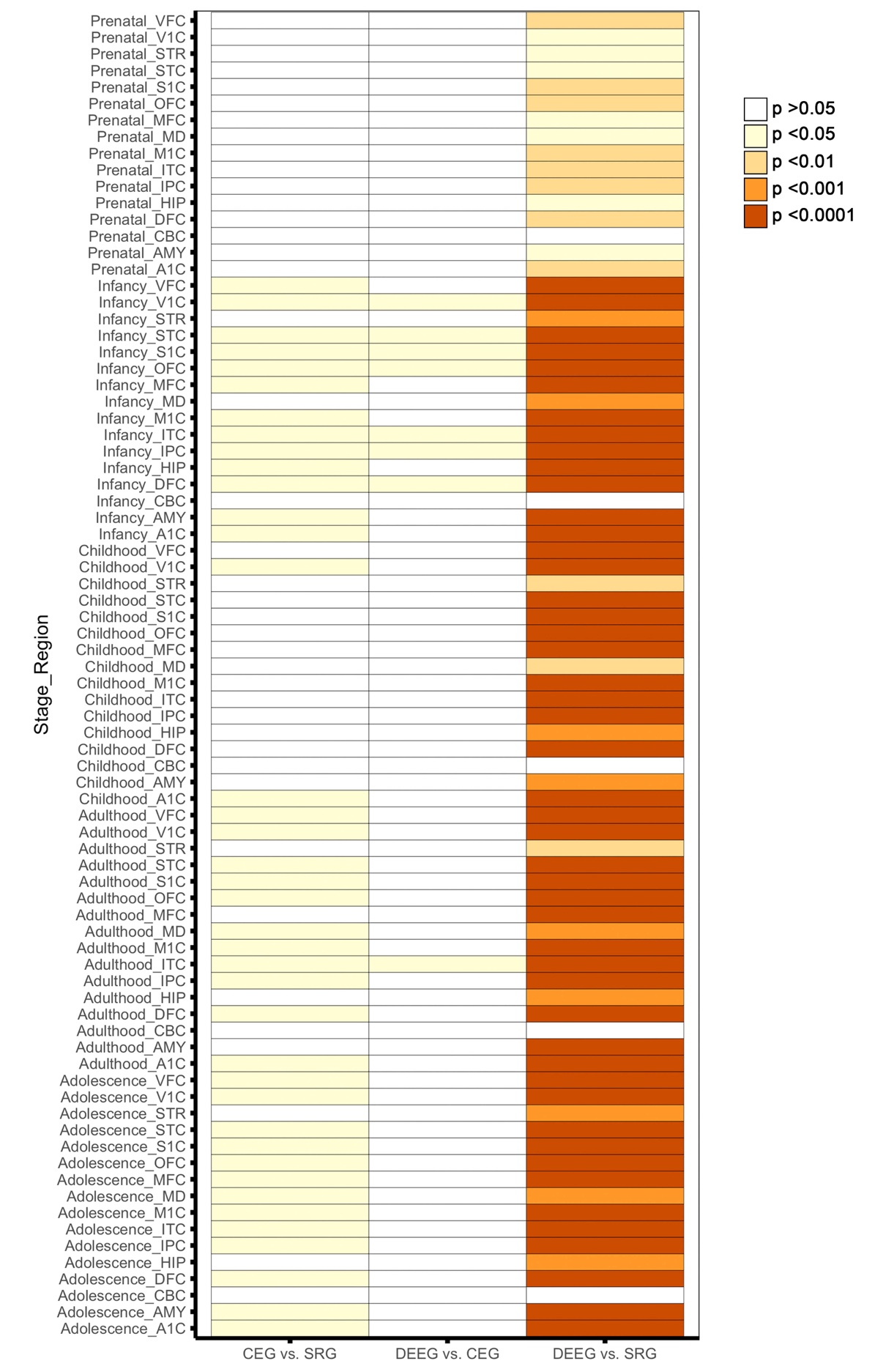
**

**Supplemental Figure 7. Heatmap of p-values from Wilcoxon signed rank sum test with Benjamini-Hochberg post hoc among the three groups of epilepsy-genes within each brain region for each developmental stage.** Bonferroni correction was applied for multiple tests across 16 different brain regions and 5 developmental periods. Significance levels are color coded.
