# Supplementary material for: Integrative analysis of epilepsy-associated genes reveals expression-phenotype correlations
Source: Sci Rep. 2024 Feb 13;14:3587. doi: 10.1038/s41598-024-53494-2 (PMC10864290; doi:10.1038/s41598-024-53494-2)
Supplement: Supplementary file 9 — Supplementary Figure 8. [file 41598_2024_53494_MOESM9_ESM.docx]

**
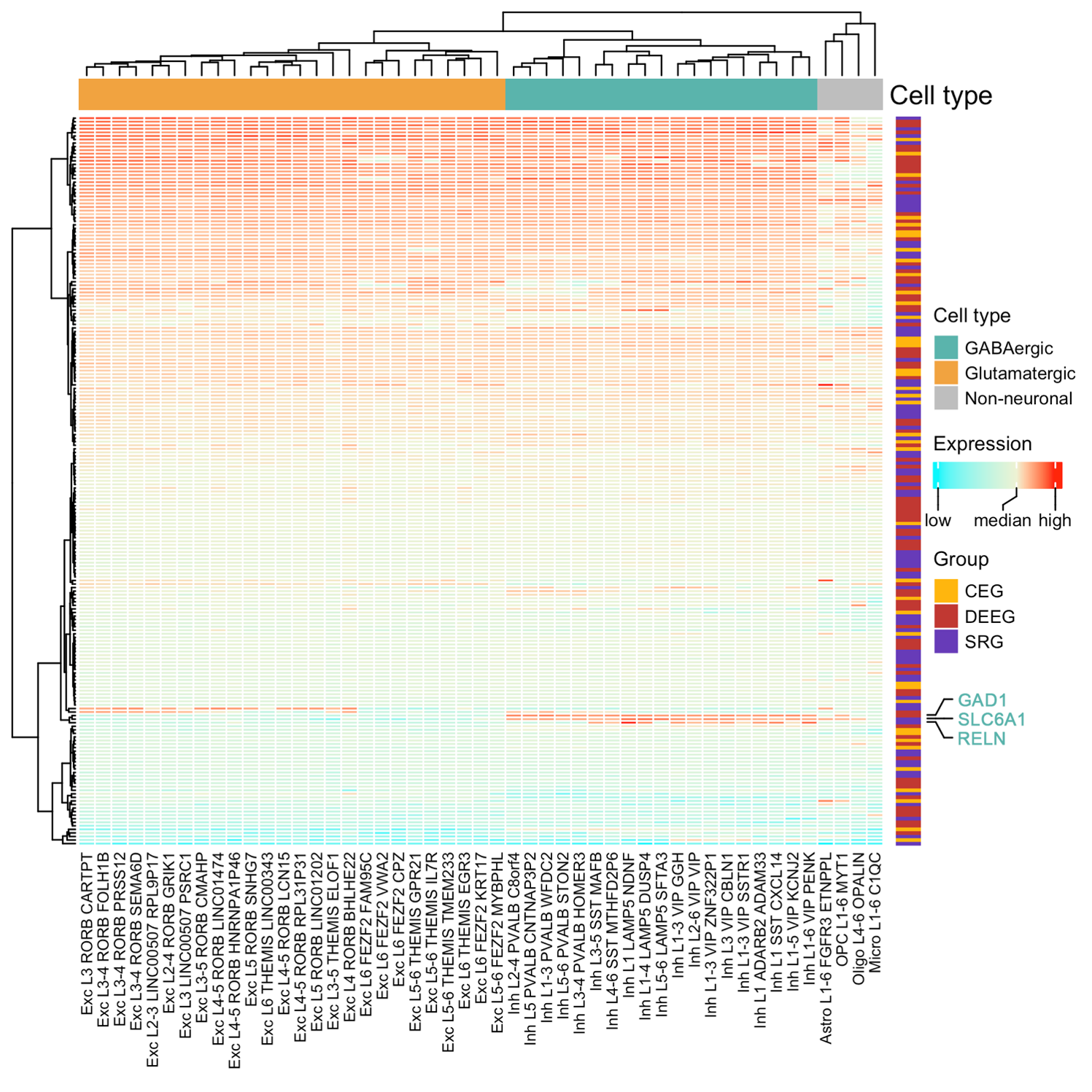
**

**Supplemental Figure 8. Averaged expression of epilepsy-associated genes in different brain cell types with hierarchical clustering across cell cluster and genes.**
